# Stakeholders' views of the implementation of pharmacist prescribing in the community pharmacy setting: a mixed-methods systematic review

**DOI:** 10.1016/j.rcsop.2026.100846

**Published:** 2026-09-19

**Authors:** Anna Colthorpe, Aoife Fleming, Suzanne McCarthy, Eimear McManus, John J Keating, Kieran Dalton

**Affiliations:** a School of Pharmacy, University College Cork, Cork, Ireland; b Pharmacy Department, Mercy University Hospital, Cork, Ireland; c School of Chemistry, University College Cork, Cork, Ireland

**Keywords:** Pharmacist prescribing, Prescription, Non-medical prescribing, Community pharmacy, Primary care, Pharmacist, Systematic review

## Abstract

**Background:**

Pharmacist prescribing in community pharmacies is gaining traction globally as a strategy to enhance medication access. However, uptake remains inconsistent, making it essential to understand stakeholder perspectives to identify factors shaping successful implementation.

**Aim:**

To synthesise the views, experiences, perceived benefits, concerns, and contextual influences of key stakeholders regarding pharmacist prescribing in community pharmacies across implementation stages.

**Method:**

A mixed-methods systematic review using a convergent integrated approach was conducted. Five databases were searched from inception to March 2025. Primary research studies were included where ≥1 key study focus was on stakeholders' views of pharmacist prescribing in community pharmacies. Thematic synthesis was used to generate themes.

**Results:**

Five analytical themes explained variation in implementation: (1) support, motivation, and clinical readiness; (2) structural, policy, and organisational conditions shaping feasibility; (3) patient-centred access, continuity, and therapeutic relationships; (4) moral responsibility, risk, and safe practice boundaries; and (5) professional identity, autonomy, and legitimacy. Overall, the literature reflected support for pharmacist prescribing, particularly for low-risk, protocol-driven conditions and collaborative care models, although views varied across stakeholder groups and contexts. Perceived benefits included improved medication access, adherence, convenience, and patient satisfaction. Concerns remained regarding accountability, medico-legal risk, care fragmentation, workload, reimbursement, and commercial conflicts of interest. Confidence, training, interprofessional collaboration, and access to records strongly influenced implementation.

**Conclusion:**

Pharmacist prescribing is shaped by interacting individual, organisational and system-level factors. Sustainable implementation requires integrated care models, workforce development, governance frameworks, and supportive policy. This review offers actionable insights for policymakers and healthcare systems seeking to integrate pharmacist prescribing into routine care.

## Highlights

• First mixed-methods review of stakeholder views on community pharmacist prescribing.
• Stakeholder acceptance was high, but implementation and uptake remained uneven.
• Confidence and readiness varied across prescribing contexts and conditions.
• Organisational and system factors influenced implementation and sustainability.
• Safe prescribing requires governance, collaboration, and integrated systems.

## 1 Introduction

Healthcare systems internationally are experiencing increasing pressure due to ageing populations, the growing prevalence of chronic disease, a rising patient demand for services, and workforce shortages.^1^, ^2^ These challenges have prompted a shift toward multidisciplinary models of care and the expansion of non-medical prescribing roles.^3^, ^4^ Within this context, community pharmacy has emerged as a key setting for healthcare delivery, with potential to improve access to treatment and alleviate pressures on other parts of the healthcare system.^5^, ^6^, ^7^ Pharmacists, with expanding clinical and prescribing responsibilities, are recognised as contributing to improved patient care and access to treatment.^5^, ^8^ Pharmacist prescribing has been introduced as a strategy to help address healthcare system pressures by improving access to care and supporting more efficient use of the healthcare workforce.^3^, ^6^

Pharmacist prescribing can have several context-dependent meanings, and its scope varies between jurisdictions.^3^, ^6^, ^7^, ^9^ For this research, pharmacist prescribing refers to the authorised initiation, modification, or discontinuation of medications via a pharmacist issuing a prescription^10^ (i.e. it is not simply recommending or supplying non-prescription medication). Models of pharmacist prescribing vary internationally and include independent prescribing, in which pharmacists assume responsibility for clinical assessment and prescribing decisions, and supplementary or collaborative prescribing models, where prescribing occurs within an agreed care plan with another prescriber.^3^, ^4^ In some jurisdictions, additional models exist, including prescribing under common conditions or minor ailment schemes, protocol-driven prescribing, and collaborative practice agreements, which enable pharmacists to initiate or manage treatment within defined clinical or regulatory frameworks.^7^, ^9^, ^11^

Over the past two decades, pharmacist prescribing has been implemented or expanded in several countries, including the United Kingdom, Canada, New Zealand, and parts of the United States.^11^, ^12^, ^13^ Introduction of these services has often been in response to healthcare system pressures such as workforce shortages and increasing demand, and as part of wider policy reforms to improve access to care.^4^, ^9^, ^12^ These developments aim to improve timely access to medicines and healthcare services, while also supporting more efficient use of the healthcare workforce.^6^, ^7^, ^9^ As a result, pharmacist prescribing is increasingly positioned as a key component of modern primary care delivery, enhancing the clinical role of pharmacists and enabling greater involvement in patient care and prescribing decision-making.^11^, ^13^

Community pharmacy represents an important setting for the implementation of pharmacist prescribing.^11^, ^13^ Community pharmacies are highly accessible, frequently used by the public, and are well positioned to support minor ailment management and self-care, with expanding roles in the management of chronic conditions and optimisation of medicines.^7^, ^14^, ^15^, ^16^ Their widespread geographical distribution, extended opening hours, and established relationships with patients position community pharmacies as a valuable setting for expanding prescribing services.^9^, ^17^

The integration of prescribing within community pharmacy has the potential to improve access to care, enable earlier intervention, and support more efficient use of healthcare resources.^9^ However, implementation in this setting differs from other healthcare contexts and presents unique organisational, professional, and operational challenges.^8^, ^18^, ^19^, ^20^ Community pharmacies often operate within retail-oriented environments, manage high dispensing workloads, and may function outside of fully integrated primary care teams.^7^, ^19^, ^20^ These contextual factors are likely to influence how prescribing roles are adopted and sustained in practice.

Implementation of pharmacist prescribing extends beyond the development of individual clinical competence and requires changes at service, organisational, and system levels.^21^ Introducing pharmacist prescribing in the community pharmacy may therefore involve workforce development, governance arrangements, workflow redesign, interprofessional collaboration, and integration with existing care pathways. Stakeholder perspectives are widely recognised as central to the implementation of new healthcare roles and services.^22^, ^23^ Pharmacists, patients, general practitioners, policymakers, regulators, and employers each play a role in shaping how prescribing is introduced, accepted, and sustained in community pharmacy.^6^, ^18^ Factors such as perceived role legitimacy, trust, role clarity and interprofessional relationships, service feasibility, and patient acceptability can influence implementation outcomes.^18^, ^24^ Understanding these perspectives is therefore essential for identifying barriers and facilitators and informing the development and implementation of effective prescribing services.

A growing body of research has explored stakeholder attitudes and experiences relating to pharmacist prescribing across a range of healthcare settings.^6^, ^16^, ^18^ Studies have reported perceived benefits of pharmacist prescribing, including improved access to care and medicines, alongside concerns relating to training, workload, governance, and interprofessional relationships.^9^, ^19^, ^20^ Patient acceptability and professional support have also been explored, with variation reported across countries and practice environments.^5^, ^25^ However, this evidence is dispersed across qualitative and quantitative studies and spans multiple healthcare contexts, including hospital, primary care, and community pharmacy settings.^13^, ^16^ As a result, the factors influencing implementation of pharmacist prescribing in community pharmacy are not always clearly identifiable.

While existing reviews have scoped out the community pharmacist role in independent prescribing (i.e. not necessarily as part of other dependent prescribing models) or examined views of pharmacist prescribing more widely in primary care,^6^, ^13^ evidence relating specifically to implementation in community pharmacy remains fragmented. A structured synthesis focusing on community pharmacy is needed to consolidate existing knowledge and provide a clearer understanding of how pharmacist prescribing is perceived and implemented in this setting. Such evidence may support policy development, service design, and future research aimed at optimising pharmacist prescribing initiatives.

This review therefore aimed to synthesise evidence on stakeholders' perspectives relating to factors influencing the implementation of pharmacist prescribing in community pharmacy settings. The objectives were to i) identify stakeholder groups represented in the literature, ii) examine reported barriers and facilitators, iii) explore stakeholder views, attitudes, and experiences related to implementation within international community pharmacy contexts where pharmacist prescribing is authorised or being considered for implementation, and, where possible, iv) compare perspectives across different stages of implementation, ultimately helping to inform policy, service development, and future research.

## 2 Methods

A mixed-methods systematic review was conducted to capture the full range of quantitative and qualitative data relevant to the research questions. A mixed-methods approach is particularly suited to examining implementation, which encompasses clinical, organisational, behavioural, and social dimensions.^22^ Quantitative studies can identify patterns in attitudes, uptake, and reported outcomes, while qualitative research provides insight into experiences, perceptions, and contextual influences.^23^ A protocol was developed following the Joanna Briggs Institute (JBI) convergent integrated approach for mixed-methods systematic reviews^26^, reported in accordance with PRISMA-P,^27^ and prospectively registered with PROSPERO (https://www.crd.york.ac.uk/PROSPERO/view/CRD42025646376). Review reporting followed JBI guidance for mixed-methods systematic reviews and the PRISMA 2020 reporting guideline.^28^

### 2.1 Search strategy

A comprehensive search of five electronic databases (PubMed, Embase, CINAHL, Web of Science, and Scopus) was undertaken on 3rd March 2025 from database inception to the search date. Search strategies were informed by an initial exploratory search to identify relevant terminology, indexing, and methodological approaches, and then iteratively refined and adapted to the requirements of each database. To reduce reporting bias, the search strategy was developed and reported in line with the Preferred Reporting Items for Systematic Reviews and Meta-Analyses literature search extension checklist (PRISMA-S),^29^ with full details provided in Appendix 2.

No publication date limits were applied to maximise search comprehensiveness. Searches were restricted to articles available in full text and published in English. Forward and backward citation searching of all included articles was performed on 26th June 2025, using the online tool ‘Citationchaser’^30^ to help potentially identify other eligible studies.

### 2.2 Eligibility criteria

Eligible studies included primary research employing quantitative, qualitative, mixed-methods, or case study designs examining factors influencing the implementation of pharmacist prescribing in community pharmacy settings internationally. Studies were included only where at least one of the study's key focuses was on the views and/or experiences of stakeholders on pharmacist prescribing in the community pharmacy setting.

Populations of interest included any stakeholders affected by or engaged with the development or delivery of pharmacist prescribing services, such as pharmacists, pharmacy staff, pharmacy students, managers, general practitioners (or international equivalents), other healthcare professionals, patients, and members of the public. Eligible contexts were community pharmacy settings internationally where pharmacists had prescribing authority through legislative, regulatory, or service frameworks, including independent, supplementary, collaborative, or common-conditions models, or where the introduction of such authority was being considered. Studies from any country were eligible where the reported study context met these criteria (i.e. countries were not selected a priori). Eligibility was determined by the nature of the prescribing authority described within the study context relative to the research team's description of pharmacist prescribing.

The primary outcomes of interest were the factors influencing the implementation of pharmacist prescribing, including barriers and facilitators across each implementation phase, informed by stakeholders' views and experiences of these services. These influences were analysed at the pre-, peri-, and post-implementation stages, referring to before, during, and after the introduction of pharmacist prescribing services.

Studies were excluded if they reported prescribing by mixed groups of healthcare professionals when data relating specifically to pharmacist prescribing could not be extracted or if they focused solely on secondary care, or mixed primary-secondary care settings where community pharmacy-specific data could not be isolated.

### 2.3 Study selection

All citations retrieved from database searches were imported into Covidence®,^31^ where duplicates were automatically removed. Two reviewers (AC and EM) independently screened titles and abstracts against the eligibility criteria. Full-text articles for all potentially eligible studies were then obtained and assessed independently by both reviewers.

Discrepancies at either stage were resolved through discussion, and if necessary through consultation with at least one other team member. Reasons for exclusion at the full-text stage were recorded.

### 2.4 Quality assessment

Quality assessment of the included studies was conducted using the Mixed Methods Appraisal Tool (MMAT).^32^ The quality assessment results did not determine study inclusion or exclusion; instead, they were used to inform interpretation of findings. A sensitivity analysis was undertaken by comparing findings across studies with differing methodological strengths, examining whether the inclusion of studies with methodological limitations influenced the overall synthesis.

### 2.5 Data extraction

Data extraction was undertaken in Covidence® using a pre-piloted extraction form created by the research team. AC extracted data from all included studies, and a 10% sample, including representation from each study type, was independently extracted by EM to ensure consistency and reliability. Extracted data included: author(s), year of publication, population, context, timeline stage (pre-, peri-, post-implementation), prescribing type, study methodology, views, and outcomes relevant to the research questions.

### 2.6 Data analysis and synthesis

A convergent integrated approach was used to synthesise qualitative and qualitised quantitative data.^26^ Quantitative findings were transformed into qualitative textual descriptions to enable integration, in accordance with JBI guidance.^26^ All data were then synthesised thematically. Thematic synthesis was selected due to its suitability for mixed-methods reviews and its ability to accommodate contextual variation and stakeholder diversity.^33^ An inductive approach was used, rather than a framework-based analysis, to avoid restricting findings to predefined categories and to better reflect the range of stakeholder views. The synthesis followed Thomas and Harden's three stages: line-by-line coding, development of descriptive themes, and generation of analytical themes.^34^

All extracted qualitative data and qualitised quantitative data were imported into NVivo®15^35^ and coded inductively using initial non-hierarchical codes. These codes were compared across studies and grouped to create an initial set of descriptive themes. An additional unclassified category temporarily held data that did not clearly fit within these initial themes. In the second stage of synthesis, the unclassified data were examined in detail to determine whether they represented new descriptive concepts or interpretive material relevant to higher-order theme development.

A remaining subset of excerpts within the unclassified category did not describe influences on implementation directly but instead revealed belief systems, value justifications, identity reasoning, moral boundaries, motivations, or hesitancy. These were grouped into a bundle to be reviewed separately and used, in line with Stage 3 of Thomas and Harden's approach,^34^ to explain the mechanisms underlying the descriptive patterns. They provided the conceptual bridge that facilitated the transformation of descriptive summaries into higher-order analytical themes.

In the final stage, the full set of descriptive themes, supported by the interpretive material, were further interpreted in relation to the review questions and contextual information to produce analytical themes which captured the mechanisms shaping the implementation of pharmacist prescribing. Analytical themes were refined through repeated review of the coded data and discussion with the wider research team. Coding and initial code categorisation were undertaken by the primary researcher (AC). Code categorisation, theme development, and theme refinement were iteratively reviewed and discussed with all research team members before theme finalisation.

### 2.7 Implementation stage classification and subgroup analysis

To explore how implementation factors varied over time, studies were given an overall categorisation into pre-, peri-, and post-implementation phases. As some studies included findings from more than one stage, each study was assigned to a single overall category based on the latest stage described. While this may have simplified studies spanning multiple phases and influenced study distribution across stages, this provided mutually exclusive categories for study-level comparison and avoided double-counting studies across stages, consistent with guidance from the Cochrane Handbook on defining groups for synthesis.^36^ However, thematic coding was conducted at the level of individual excerpts, allowing findings relating to different implementation stages within the same study to be retained. Each excerpt was coded to the implementation stage to which it related. Where the same issue appeared at different stages, it was coded separately for each stage. This approach enabled comparison of themes across stages.

Themes were compared across implementation phases to explore how barriers and facilitators were described at different stages of implementation. Stakeholder perspectives were also considered during the synthesis, with variations and conflicting perspectives between stakeholder groups reported where identified. Theme occurrence was not formally quantified or compared between stakeholder groups.

### 2.8 Rigour and reflexivity

Rigour was enhanced through use of a pre-registered protocol, maintenance of an audit trail (Covidence®, NVivo®, Microsoft® Excel records), and reflexive team discussions. The research team comprised pharmacists with both clinical and academic expertise: AC and EM are pharmacists with extensive clinical experience (AC as an independent prescriber with experience of prescribing in secondary care settings and EM as an advanced specialist pharmacist in infectious diseases), while the remaining authors are pharmacists and academics with expertise in evidence synthesis and clinical practice. These different professional perspectives were considered throughout coding and synthesis to ensure that the findings were grounded in the data and reflected the various stakeholder views. Coding was completed by the primary reviewer (AC) and discussed regularly with the wider team to support balanced interpretation and reduce individual bias.

## 3 Results

### 3.1 Search results

The original database search yielded 5633 papers, with 3309 studies remaining for screening after duplicate removal. Citation searching did not identify any additional eligible studies. The flow of studies is shown in Fig. 1, with 78 included in the review.

**Fig. 1:**
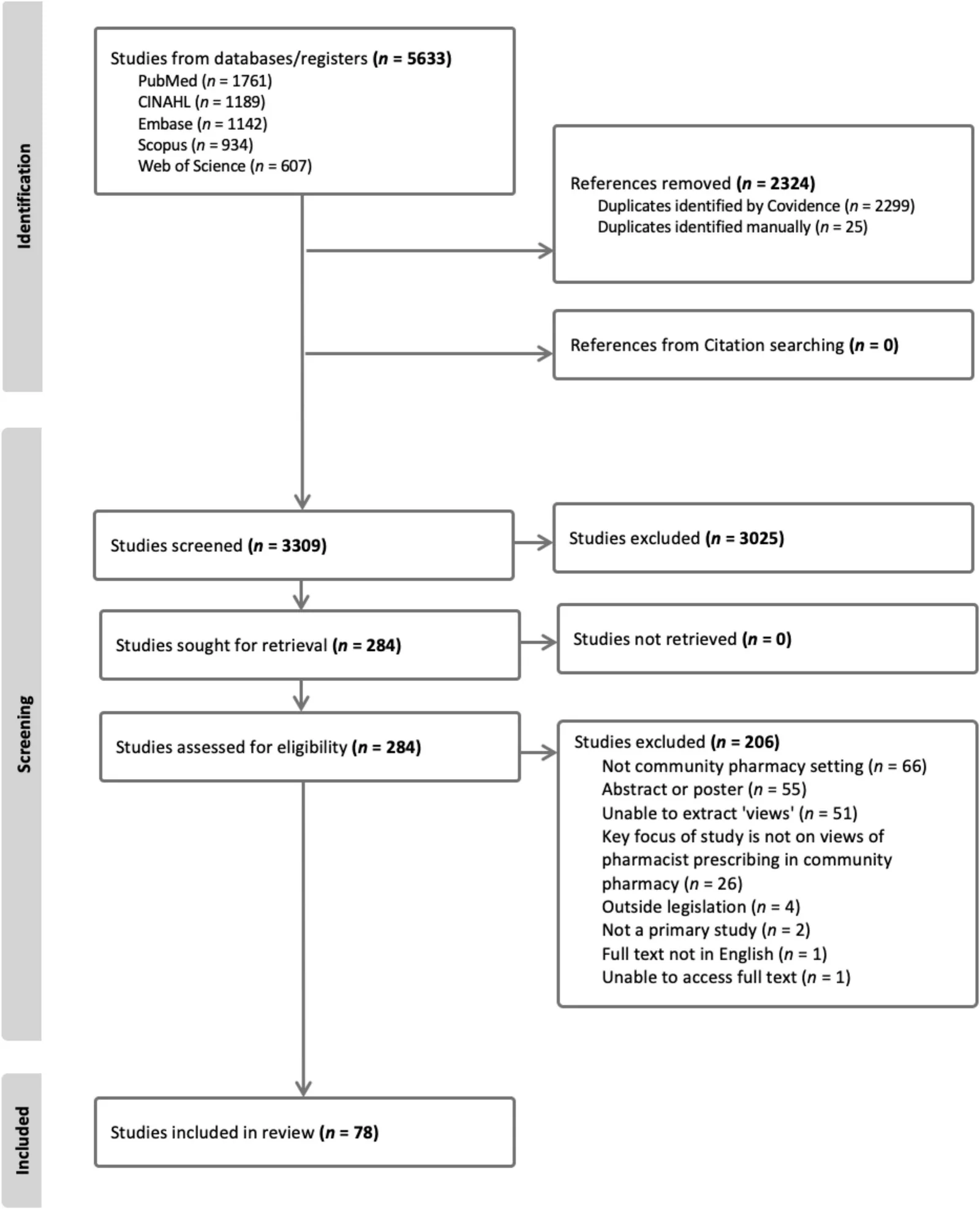
PRISMA flow diagram.

### 3.2 Characteristics of included studies

Table 1 provides a synthesised summary of included studies' characteristics, while Table 2 displays these characteristics individually for each study. Over one third of studies originated from the United States (*n* = 30, 38.4%), followed by approximately one fifth from Canada (*n* = 16, 20.5%) and approximately one eighth from the United Kingdom (*n* = 10, 12.8%). Quantitative study designs were most common (*n* = 46, 59.0%), accounting for nearly two thirds of included studies, while nearly one third used qualitative methods only (*n* = 23, 29.5%) and nine studies employed mixed methods (11.5%). Two thirds of studies included one stakeholder type (*n* = 52), while one third had mixed stakeholder groups (*n* = 26). Community pharmacists were the most frequently studied stakeholder group (*n* = 55, 70.5%). The most common study focus was non-specific or multi-condition prescribing (*n* = 38, 48.7%), followed by hormonal contraception-related prescribing (*n* = 25, 32.1%). Government protocol prescribing (*n* = 48, 61.5%) and independent prescribing (*n* = 32, 41.0%) were the most commonly discussed prescribing models. Over half of studies were published between 2020 and 2025 (*n* = 42, 53.8%).

**Table 1:** Summary of included studies' characteristics.

Characteristic and Category	Number of studies (*n* = 78)	Percentage %
**Country**		
United States	30	38.4
Canada	16	20.5
United Kingdom	10	12.8
Australia	5	6.4
South Africa	3	3.8
Malta	2	2.6
New Zealand	2	2.6
Nigeria	2	2.6
Switzerland	2	2.6
France	1	1.3
Ireland	1	1.3
Italy	1	1.3
Jordan	1	1.3
Poland	1	1.3
United Arab Emirates	1	1.3

**Study type**		
Quantitative	46	59
Qualitative	23	29.5
Mixed methods	9	11.5

**Main study focus**†		
Non-specific / multiple-condition prescribing	38	48.7
Hormonal / emergency contraception / medical abortion	25	32.1
Antibiotics	5	6.4
PrEP / PEP (HIV) ⁎	5	6.4
Common conditions / minor ailments	4	5.1
Naloxone	1	1.3
Oral anticoagulants	1	1.3

**Stakeholders**†		
Community pharmacist	55	70.5
Pharmacist (other sectors)	18	23.1
Patients / service users	14	17.9
General practitioner (GP)	11	14.1
Other (e.g., commissioners, policy makers)	10	12.8
General public	8	10.3
Pharmacy student	6	7.7
Doctor (not GP)	3	3.8

**Prescribing type(s) discussed**†		
Government protocol	48	61.5
Independent prescribing	32	41
Emergency / minor ailments	25	32.1
Dependent prescribing (e.g., collaborative agreements, supplementary prescribing)	27	34.6
Repeat / continuation prescribing	15	19.2
Other	13	16.7

**Prescribing stage**†		
Pre-implementation (Pre)	39	50
Peri-implementation (Peri)	10	12.8
Post-implementation (Post)	36	46.2

**Year of publication**		
1990–1999	1	1.3
2000–2009	6	7.7
2010–2019	29	37.2
2020–2025 ∆	42	53.8

⁎ PrEP: Pre-exposure prophylaxis; PEP: Post-exposure prophylaxis; HIV: human immunodeficiency virus.

† Categories may exceed 100% because some studies included multiple stakeholders or prescribing models or stages.

∆ Up until March 2025 included.

**Table 2:** Characteristics of included studies.

Author, Year	Country; Implementation Stage ⁎	Study Aim(s)	Stakeholder type(s)	Study Design; Data Collection
Amador-Fernández et al. 2023 ^37^	Switzerland; Post	To identify implementation factors and to evaluate pharmacy association interventions that aim to support implementation of pharmacist prescribing in Switzerland.	Community Pharmacist; Board of Pharmacy Associations	Qualitative; Interviews, Questionnaire
Amador-Fernández et al. 2024 ^38^	Switzerland; Post	To evaluate the clinical relevance of pharmacist-prescribed medications compared to the over-the-counter medications and to evaluate its implementation into daily practice.	Community Pharmacist	Mixed Methods; Survey
Attard Pizzuto et al. 2019 ^39^	Malta; Pre	To investigate the perception of Maltese pharmacists to prescribe a selected number of antibiotics.	Community Pharmacist	Quantitative; Questionnaire
Auta et al. 2014 ^40^	Nigeria; Pre	To explore the views of patients of community pharmacists on their consultation experiences, and the possible extension of prescribing rights to pharmacists in Nigeria.	Patient	Qualitative; Questionnaire
Auta et al. 2016 ^41^	Nigeria; Pre	To investigate stakeholders' views on granting prescribing authority to pharmacists in Nigeria.	Community Pharmacist; Pharmacist (other); GP; Doctor (not GP)	Qualitative; Interviews
Azad et al. 2024 ^42^	United States; Post	To quantify the extent of pharmacists' furnishing (prescribing) of hormonal contraception in California's Central Valley community pharmacies and identify barriers and facilitators to implementation.	Community Pharmacist	Mixed Methods Interviews; Survey
Banawis et al. 2023 ^43^	United States; Post	To assess pharmacist furnishing rates of naloxone in California's Central Valley and identify barriers and facilitators to implementation.	Community Pharmacist	Mixed Methods Interviews; Survey
Baratta et al. 2025 ^44^	Italy; Pre	To investigate the attitudes of pharmacists, family doctors, and pharmacy users in North-West Italy toward the possible introduction of pharmacist prescribing, and to identify expected benefits, risks, and training needs.	Community Pharmacist; GP; Patient; General Public	Quantitative Interviews; Questionnaire
Beahm et al. 2018 ^45^	Canada; Post	To evaluate the effectiveness, safety, and patient satisfaction with pharmacist prescribing and care in patients with uncomplicated urinary tract infections.	Community Pharmacist; Patient	Quantitative; Questionnaire
Booker et al. 2023 ^46^	Canada; Pre	To determine community pharmacists' acceptance of a pharmacist pre-exposure prophylaxis (PrEP) prescribing service in Nova Scotia and identify barriers and facilitators using the Theoretical Framework of Acceptability.	Community Pharmacist	Mixed Methods; Interviews, Survey
Brilliant Moloto et al. 2024 ^47^	South Africa; Peri	To explore the barriers to community pharmacists' prescribing role in Limpopo Province and provide recommendations to enhance pharmacist prescribing at community pharmacies.	Community Pharmacist	Qualitative; Interviews
Campi et al. 2024 ^48^	United States; Post	To gain insight into successful implementation of pharmacist-prescribed contraceptive services by Interviewing early adopters, focusing on their approaches, successes, challenges, lessons learned, and implications for practice.	Community Pharmacist	Qualitative; Interviews
Chuene et al. 2024 ^49^	South Africa; Post (Peri, Post)	To explore and describe community pharmacists' perceptions and attitudes toward their emerging role in prescribing within community pharmacies in Polokwane, Limpopo province, South Africa.	Community Pharmacist	Qualitative; Interviews
Cohen et al. 2024 ^50^	United States; Pre	To explore the feasibility of broadening pharmacist scope of practice in California to include the prescribing of medication for abortion, by examining pharmacist willingness, knowledge, confidence, and perceived barriers.	Community Pharmacist	Quantitative; Survey
Dahri et al. 2024 ^51^	Canada; Post	To gather community-based pharmacists' perspectives in British Columbia related to prescribing for minor ailments and contraception.	Community Pharmacist	Quantitative; Survey
Dale et al. 2024 ^52^	United States; Post (Pre, Peri, Post)	To understand the state-level steps to enable pharmacist prescribing of hormonal contraception, including implementation challenges and facilitators.	Community Pharmacist; Pharmacist (other); Pharmacist association, Medicaid officials	Qualitative; Interviews
Famiyeh et al. 2019 ^53^	Canada; Post	To describe Ontario community pharmacy service users' support for and stated willingness to use pharmacist prescribing services.	Patient	Qualitative; Interviews
Faruquee et al. 2019 ^54^	Canada; Post	To explore Albertan family physicians' perceptions and relational dynamics surrounding pharmacists' prescribing practices.	GP	Qualitative; Interviews
Feehan et al. 2016 ^55^	United States; Pre	To explore the perceived demand for and barriers to pharmacist prescribing in the community pharmacy setting.	Community Pharmacist; Patient; Reimbursement decision-makers	Qualitative; Interviews
George et al. 2006 ^56^	United Kingdom; Pre	To investigate Scottish community pharmacists' awareness relating to the consultation on independent prescribing by pharmacists, their perceptions of competence and training needs for the management of selected common conditions relevant to community pharmacy practice, their views and attitudes toward patient safety and accessibility of independent prescribing by community pharmacists, and to identify factors associated with those attitudes.	Community Pharmacist	Quantitative; Survey, Questionnaire
George et al. 2007 ^57^	United Kingdom; Post	To investigate the challenges experienced by pharmacists in Great Britain in delivering supplementary prescribing (SP) services, to explore their perceptions of benefits of SP and to obtain feedback on both SP training and implementation.	Pharmacist (other)	Quantitative; Questionnaire
Gomez et al. 2020 ^58^	United States; Peri	To identify barriers and facilitators to offering pharmacist-prescribed hormonal contraception in Californian independent pharmacies.	Community Pharmacist	Qualitative; Interviews
Gomez et al. 2022 ^59^	United States; Post	To describe awareness, interest, acceptability, and support for pharmacist-prescribed contraception in a rural California county.	Patient; General Public	Quantitative; Survey
Grant et al. 2023 ^60^	Canada; Post	To determine the extent of self-reported pharmacist prescribing pre COVID-19 and during the COVID-19 pandemic, to identify barriers and facilitators to pharmacist prescribing, and to explore the relationship between these factors and self-reported prescribing activity.	Community Pharmacist	Mixed Methods; Survey, Questionnaire
Grant et al. 2025 ^61^	Canada; Post	To describe barriers and facilitators to pharmacist prescribing and their impact on self-reported prescribing activity and to identify potential behavioural change interventions based on the identified barriers and facilitators.	Community Pharmacist	Mixed Methods; Interviews, Survey
Guirguis et al. 2014 ^62^	Canada; Post	To explore and characterise how pharmacists in Alberta have integrated prescribing privileges into practice across different settings, three years after legislation was enacted.	Community Pharmacist; Pharmacist (other)	Qualitative; Interviews
Hatah et al. 2013 ^63^	New Zealand; Pre	To evaluate general practitioners' (GPs') perceptions of pharmacists' contributions to tasks traditionally undertaken by GPs such as medication review and prescribing.	GP	Qualitative; Interviews
Herman et al. 2020 ^64^	United States; Post	To explore pharmacist perspectives on prescribing hormonal contraceptives, including perceived barriers and facilitators to implementation in rural New Mexico and opinions on over-the-counter access, and prescribing and inserting subdermal contraceptive implants.	Community Pharmacist	Qualitative; Interviews
Hoti et al. 2011 ^65^	Australia; Pre	To examine the views of regular pharmacy clients on pharmacist prescribing and employ agency theory to consider the relationships between stakeholders.	Patient	Quantitative; Interviews, Survey, Questionnaire
Hoti et al. 2013 ^66^	Australia; Pre	To compare attitudes of Australian hospital and community pharmacists in relation to pharmacist prescribing with emphasis on pharmacist prescribing models, reasons in favour of and barriers to assuming such prescribing roles, as well as preferred therapeutic areas of prescribing.	Community Pharmacist; Pharmacist (other)	Quantitative; Questionnaire
Hughes et al. 2003 ^67^	United Kingdom; Pre	To explore perceived barriers between GPs and community pharmacists in relation to closer interprofessional working and the extension of prescribing rights to pharmacists.	Community Pharmacist; GP	Qualitative; Focus Group
Hughes et al. 2014 ^68^	Canada; Post	To explore what “prescribing” means to pharmacists in Alberta and how prescribing is applied in practice.	Community Pharmacist; Pharmacist (other)	Qualitative; Interviews
Hunter et al. 2023 ^69^	United States; Post	To evaluate and inform the implementation of California's Senate Bill, which permitted pharmacists to independently prescribe PrEP and post exposure prophylaxis (PEP), by assessing knowledge, attitudes, practices, barriers, and preferences among California pharmacists and pharmacy students.	Pharmacy student; Community Pharmacist; Pharmacist (other)	Quantitative; Survey
Irwin et al. 2019 ^70^	United States; Post	To characterise public perceptions of pharmacist-prescribed self-administered non-emergency hormonal contraception using online news article comments.	General Public	Mixed Methods; Online comments (retrospective social media data)
Karim et al. 2024 ^71^	United Kingdom; Post	To use Normalization Process Theory to investigate the integration of pharmacist independent prescribing in community pharmacy in the context of NHS Pharmacy First Plus in Scotland.	Community Pharmacist; Pharmacist (other)	Quantitative; Survey,Questionnaire
Koester et al. 2020 ^72^	United States; Pre	To assess key stakeholder attitudes about California legislation allowing community pharmacists to furnish human immunodeficiency virus (HIV) PrEP and PEP.	Pharmacy student; Pharmacist (other); Doctor (not GP); Pharmacy chain representatives	Qualitative; Interviews
Lemay et al. 2025 ^73^	United States; Pre	To assess college student and community pharmacy patient perspectives on pharmacist-prescribed hormonal contraceptives, likelihood of accessing contraceptives from a pharmacist, and views on emergency contraception and over-the-counter oral contraception.	Pharmacy student; Patient	Qualitative; Survey, Questionnaire
Lewis et al. 2021 ^74^	Canada; Pre	To evaluate attitudes, beliefs, and perceptions of independent pharmacist prescribing among community and health authority pharmacists in northern British Columbia, and to assess their likelihood of applying for prescribing authority if it were to become available.	Community Pharmacist; Pharmacist (other)	Quantitative; Survey, Questionnaire
Lio et al. 2018 ^75^	United States; Peri (Pre; Peri)	To compare community pharmacists' comfort levels and knowledge about prescribing hormonal contraception before and after a training session and to identify perceived barriers and resources needed	Community Pharmacist	Quantitative; Survey
Lloyd et al. 2007 ^76^	United Kingdom; Pre	To explore the views and professional context of pharmacists and their physician mentors regarding pharmacist supplementary prescribing, prior to training.	Community Pharmacist; Pharmacist (other); GP; Doctor (not GP)	Qualitative; Interviews, Focus Group
Lutz et al. 2021 ^77^	United States; Peri	To assess patient perspectives of pharmacist PrEP prescribing and identify potential barriers to acceptance.	Patient	Quantitative; Survey, Questionnaire
Makowsky et al. 2013 ^78^	Canada; Post	To understand factors influencing pharmacists' adoption of prescribing, using Greenhalgh's Diffusion of Innovations model.	Community Pharmacist; Pharmacist (other)	Qualitative; Interviews
Mansell et al. 2015 ^79^	Canada; Post	To determine whether patients prescribed treatment for minor ailments by a pharmacist symptomatically improve within a set time frame.	Patient	Quantitative; Questionnaire
Mantzourani et al. 2023 ^80^	United Kingdom; Peri	To explore the experiences of independent pharmacist prescribers and NHS commissioners regarding the new Independent Prescribers' Service in Wales.	Community Pharmacist; NHS Commissioners	Qualitative; Interviews
Meredith et al. 2020 ^81^	United States; Pre	To identify how women at risk for unintended pregnancy in central Indiana perceive pharmacist prescribing of contraception.	Patient	Qualitative; Interviews
Mhailan et al. 2022 ^82^	Jordan; Pre	To assess community pharmacists' attitudes toward expanding their prescribing role and to identify contributing factors, preferred models, barriers, and training needs.	Community Pharmacist	Quantitative; Questionnaire
Miszewska et al. 2022 ^83^	Poland; Post	To determine current practice of pharmaceutical prescribing in Poland and pharmacists' opinions on their new prescribing rights following recent legal changes.	Community Pharmacist; Pharmacist (other)	Quantitative; Questionnaire
Mody et al. 2019 ^84^	United States; Post	To understand knowledge, current practices, and perceived barriers/facilitators to pharmacists prescribing emergency contraception.	Community Pharmacist	Quantitative; Survey
Moore et al. 2014 ^85^	Ireland; Pre	To explore the general practitioner-pharmacist relationship, to gain insight into communication between the professions and evaluate opinion on extension of the role of the community pharmacist.	Community Pharmacist; GP	Quantitative; Questionnaire
Mospan et al. 2021 ^86^	United States; Pre	To identify student pharmacists' attitudes toward pharmacist-prescribed hormonal contraception, perceived barriers, and factors affecting their viewpoints.	Pharmacy student	Quantitative; Questionnaire
Norman et al. 2015 ^87^	Canada; Pre	To assess the acceptability and feasibility of independent prescribing of hormonal contraception by pharmacists in community pharmacies across British Columbia, Canada, and to identify perceived facilitators and barriers to implementation.	Community Pharmacist	Mixed Methods; Interviews, Survey, Questionnaire
O'Connell et al. 2020 ^88^	United States; Pre	To determine Michigan female college students' opinions and willingness to use pharmacists for obtaining hormonal contraception in a community pharmacy, including perceived advantages, concerns, and factors influencing their decision.	General Public	Quantitative; Survey
Papineau et al. 2021 ^89^	United States; Pre	To assess the perceptions and attitudes of student pharmacists in Indiana regarding pharmacist prescribing of hormonal contraceptives, including readiness, perceived benefits, barriers, and training needs.	Pharmacy student	Quantitative; Survey
Pelaccio et al. 2022 ^90^	United States; Pre	To analyse the impact of the COVID-19 pandemic on birth control use and access, and evaluate women's perceptions of pharmacist-prescribed birth control.	Patient; General Public	Quantitative; Survey
Perraudin et al. 2011 ^91^	France; Pre	To analyse the opinions of final-year pharmacy students on expanding the scope of pharmacists' practices in France and thereby assess the potential barriers to adopting new practices.	Pharmacy student	Quantitative; Questionnaire
Rafie et al. 2019 ^92^	United States; Pre	To explore the outlooks of community pharmacists in California regarding prescribing hormonal contraception after the first state legislation expanding pharmacist scope of practice, but prior to full state-wide protocol implementation.	Community Pharmacist	Qualitative; Interviews
Rafie et al. 2022 (1)^93^	United States; Post	To describe patient experiences using pharmacist-prescribed hormonal contraception in California community pharmacies, focusing on acceptability, satisfaction, and preventive care behaviours.	Patient; General Public	Quantitative; Survey
Rafie et al. 2022 (2)^94^	United States; Post	To determine barriers and facilitators for pharmacists prescribing emergency contraception under California's state-wide protocol.	Community Pharmacist; Pharmacist (other); Pharmacy technicians	Qualitative; Interviews
Raghunandan *et al.* 2021 ^95^	New Zealand; Post (Peri; Post)	To determine community pharmacists' preferences for prescribing services in New Zealand primary care and explore how service characteristics (e.g., prescribing model, education, remuneration, service location) influence willingness to engage in pharmacist prescribing.	Community Pharmacist	Quantitative; Questionnaire
Rashid et al. 2022 ^96^	United States; Pre	To quantify motivating factors and barriers to pharmacist-prescribed contraception in Michigan community pharmacies, and compare pharmacies interested versus not interested in implementing the service, using the Theoretical Domains Framework.	Community Pharmacist; Pharmacist (other)	Quantitative; Survey
Reyes et al. 2020 ^97^	United States; Post	To assess California community pharmacists' knowledge, motivation, barriers, and confidence regarding provision of patient care services, including prescribing under expanded scope of practice and their ability to bill for such services.	Community Pharmacist	Quantitative; Survey
Rouleau et al. 2024 ^98^	Canada; Post	To assess community pharmacists' comfort levels in adjusting medications and following up pharmacotherapy under Québec's expanded prescribing scope, and to identify barriers and facilitators to implementation.	Community Pharmacist	Quantitative; Survey
Sadeq et al. 2022 ^99^	United Arab Emirates; Pre	To evaluate the perceptions, attitudes, confidence, requirements, barriers, and perceived competence of community pharmacists in the United Arab Emirates regarding independent prescribing in community pharmacies.	Community Pharmacist	Quantitative; Survey
Sandhu et al. 2018 ^100^	Canada; Post	To evaluate the knowledge, confidence, and perceived barriers of community pharmacists regarding prescribing oral anticoagulants for atrial fibrillation in Alberta, and to assess their ability to identify eligible patients using case scenarios.	Community Pharmacist	Quantitative; Survey
Seamon et al. 2020 ^101^	United States; Peri ( Pre; Peri)	To determine the likelihood of North Carolina community pharmacists to prescribe hormonal contraception and to identify perceptions, benefits, and barriers to pharmacist-prescribed contraception across community and non-community settings.	Community Pharmacist; Pharmacist (other)	Quantitative; Survey
Sinkala et al. 2018 ^102^	Australia; Pre	To examine community pharmacists' support for rescheduling (“*down-scheduling*”) selected oral antibiotics, and to evaluate their decision-making and appropriateness of prescribing for common infections through case vignettes.	Community Pharmacist	Quantitative; Survey (case vignettes)
Spencer et al. 1992 ^103^	United Kingdom; Pre	To ascertain United Kingdom general practitioners' attitudes toward an extended role for community pharmacists, including prescribing, minor illness management, and professional collaboration.	GP	Quantitative; Questionnaire
Stewart et al. 2009 ^104^	United Kingdom; Post	To determine the awareness, views, and attitudes of the Scottish general public toward nonmedical prescribing, with a particular emphasis on pharmacist prescribing.	General Public	Quantitative; Survey
Stewart et al. 2018 ^105^	United Kingdom; Post	To evaluate the implementation of the Pharmacy First service in Forth Valley (Scotland) for community pharmacist management of uncomplicated urinary tract infections, impetigo, and chronic obstructive pulmonary disease exacerbation, focusing on patient access, GP workload, referral appropriateness, and stakeholder feedback.	Community Pharmacist; GP; Patient; Reception Staff	Quantitative; Questionnaire
Stone et al. 2024 ^106^	United States; Pre	To compare perceptions of community and non-community pharmacists in Georgia regarding pharmacist-prescribed hormonal contraception, with secondary aims to evaluate community pharmacists' interest, comfort, and barriers, and differences between metropolitan vs. nonmetropolitan areas.	Community Pharmacist; Pharmacist (other)	Quantitative; Survey
Truter et al. 2018 ^107^	South Africa; Pre	To determine the perceptions of community pharmacists toward prescribing antibiotics and their views on antibiotic use in primary care in Nelson Mandela Bay, South Africa.	Community Pharmacist	Quantitative; Questionnaire
Ung et al. 2017 ^108^	Australia; Pre	To explore community pharmacists' readiness to prescribe oral antibiotics for limited infections, focusing on their confidence, appropriateness of prescribing, and support for expanded prescribing roles.	Community Pharmacist	Quantitative; Questionnaire (case vignettes)
Vracar et al. 2008 ^109^	Australia; Pre	To explore Australian general practitioners' views on extending prescribing rights to pharmacists, the appropriateness of pharmacist prescribing models, and the influence of GPs' characteristics on their preference for a particular pharmacist prescribing model.	GP	Mixed Methods; Interviews, Questionnaire
Vu et al. 2019 ^110^	United States; Pre	To assess California pharmacists' intentions to prescribe hormonal contraception following new legislative authority, and to identify perceived motivations, barriers, comfort levels, and training needs prior to state-wide protocol implementation.	Community Pharmacist	Quantitative; Survey
Waite et al. 2018 ^111^	Canada; Post	This study sought to better understand how recent graduates perceive their preparedness for providing full-scope pharmacist services and whether specific demographic and practice characteristics affect this perception.	Community Pharmacist	Quantitative; Survey
Warner et al. 2025 ^112^	United Kingdom; Post	To explore stakeholders' attitudes toward the responsibilities and accountabilities of newly qualified pharmacist independent prescribers and to identify enablers for safe, large-scale implementation in community pharmacy and other settings.	Community Pharmacist; Pharmacist (other); GP; Policy Makers; Educators; Insurers	Qualitative; Interviews
Wirth et al. 2011 ^113^	Malta; Pre	To determine the perception of Maltese community pharmacists regarding supplementary prescribing, including suitable conditions, perceived barriers, and requirements for implementation.	Community Pharmacist	Quantitative; Questionnaire
Zhu et al. 2020 ^114^	United States; Post (Peri; Post)	To determine patients' perceptions of pharmacist-prescribed PrEP, including specifically determining whether perceptions differed between patients who had previously been prescribed PrEP versus those who had not, and between those who had at least one indication for PrEP versus those who did not have any indications.	General Public	Quantitative Survey

Abbreviations: COVID-19: coronavirus disease 2019; GP: general practitioner; HIV: human immunodeficiency virus; NHS: National Health Service; PEP: post-exposure prophylaxis; PrEP: pre-exposure prophylaxis; SP: supplementary prescribing.

⁎ Implementation stage: As some studies reported findings across more than one stage, each was assigned to a single overall category based on the latest stage described to support subgroup comparison.

### 3.3 Quality of included studies

#### 3.3.1 The MMAT quality assessment results are shown in Appendix 5

Qualitative studies were of uniformly high methodological quality, as all studies met all MMAT criteria. In contrast, quantitative descriptive studies and the quantitative components of mixed-methods studies exhibited several methodological limitations; while generally employing appropriate measurement tools and analytical approaches, many studies relied on convenience sampling or voluntary survey participation, resulting in lower ratings for sample representativeness. Similarly, risk of nonresponse bias was frequently rated as high or unclear due to low response rates or limited reporting regarding non-responders. Mixed-methods studies generally met most MMAT criteria and demonstrated appropriate integration of qualitative and quantitative components, although the quantitative elements shared similar sampling limitations.

Sensitivity analysis indicated that excluding studies with greater methodological limitations did not substantially alter the main themes identified. All five analytical themes remained represented after studies with greater methodological limitations were excluded, with no substantive change to interpreting the findings. Therefore, this demonstrates the robustness of the synthesised evidence.

#### 3.3.2 Descriptive and analytical themes

A total of five overarching analytical themes, comprising a total of twenty descriptive subthemes, were generated from the data. These themes reflect the key factors shaping implementation, feasibility, clinical practice, and professional perspectives across the included studies. A summary of the themes, together with illustrative quotations and supporting data, is presented in Table 3, while the contributions of individual studies to the synthesis are detailed in Appendix 4.

**Table 3:** Theme summaries, illustrative quotations, and supporting data.

Descriptive themes under analytical themes	Descriptive theme summary	Examples of illustrative quotations and supporting data
**1. Support, motivation, and clinical readiness**		
1.1 Support and perceived clinical appropriateness	Overall support for pharmacist prescribing was high, particularly for low-risk, protocol-driven services perceived to improve access and patient care.	“*I think under the right conditions they should be able to. If a person does not suffer any chronic illness and just needs a basic antibiotic for a urinary tract infection or sinus infection, then the pharmacist is well schooled… so let him make these calls.*”^55^ Pharmacists identified several benefits of prescribing hormonal contraception: improved access (68.8%), increased patient adherence and use through pharmacy access (69.5%).^101^
1.2 Motivations, professional aspirations, and role perceptions	Prescribing was motivated by patient-centred care, professional development opportunities, and aspirations to expand clinical roles, though engagement varied according to role perceptions, context, and incentives.	“*I've always been one that believes in putting your own belief system behind to uphold the profession… as long as it's within the patient's belief system that should be all that matters*.”^46^ “*A lot of pharmacists feel like having that extra work* […] t*here's not enough incentives for the pharmacists to go through the training and to be certified because they feel like it's additional time away from other jobs they already have to do, and they* […] *feel overburdened, that there's not enough help, there's not enough hours, and then there's not enough pay for pharmacists to be doing this as well now*.”^43^
1.3 Preparedness and confidence	Pharmacists generally felt confident prescribing within defined scopes, with confidence highest for familiar, low-complexity conditions.	“*Although I found the university section useful, I learnt so much sitting in with the GP/nurse. I feel this experience with the GP has helped me a lot, even in working in the shops*.”^57^
1.4 Knowledge and skills gaps	Gaps in clinical assessment, diagnosis, and monitoring-rather than medicines knowledge, were consistently identified as barriers to independent prescribing.	“*I would have concerns about pharmacists writing prescriptions though… I don't think they would be adequately trained… It's a dangerous approach. They're not adequately trained to recognise disease*”^55^ “*It is difficult for pharmacists to offer prescribing services when we have no formal training on how to do an assessment like a physician … there is no consistency from pharmacy to pharmacy. We were given very little notice … to do additional prescribing services (contraceptives, UTI, Shingles) and were never offered any additional training or help to take on these roles. If I want training, I have to try to find courses to take on my days off and pay for it out of pocket*”^60^
**2. Structural, policy, and organisational conditions shaping feasibility**		
2.1 Workflow and documentation	Prescribing was more feasible when workflows and documentation systems aligned with existing pharmacy services and minimised administrative burden.	“*One of the major issues in community pharmacy at this time is workload… the design of the current workflow in most community pharmacies just is not optimized to support that.*”^46^ Almost three-quarters of pharmacists (74%) found documentation straightforward, with some using patient group directions as checklists.^105^ Pharmacists estimated that about 44% of eligible expanded-scope activities were not performed due to workflow and organisational barriers.^98^
2.2 Time and staffi ng pressures	Limited time and staffing capacity constrained pharmacists' ability to integrate prescribing into routine practice.	“*The reality is that something would have to give. We can't continue to add to pharmacists' plate without something giving. Whether that's intentional or unintentional, right? so, whether you know, we intentionally limit the other services that we're able to provide in a specific time frame or whether unintentionally the quality of our other work suffers due to time constraints and workflow issues*.”^46^ “*I would like to be [trained] someday; I just haven't had the time to do it*.”^92^
2.3 Team communication and coordination	Clear communication and coordination within pharmacy teams and with other healthcare professionals facilitated prescribing uptake.	“*We're supposed to be doing team-based care… When you don't have any of those things and all you get back is faxes, you don't have team-based care; you have just another silo of primary care*.”^54^ “*I have an issue with them making changes without communication and trying to assess patients without communication and making a bunch of recommendations based on whatever they decide they're going to make and so if that's appropriate or inappropriate, how do you know*?”^54^
2.4 Clinical criteria, eligibility, and complexity	Feasibility declined as patient and clinical complexity increased, with pharmacists favouring clearly defined eligibility criteria and referral pathways.	Pharmacists demonstrated strong ability to identify patients eligible for stroke prevention, with between 55% and 92% correctly recognising eligibility.^100^ “*You're prescribing something at somebody else's diagnosis. You still need to make sure that that diagnosis is correct. You need to ask if the initial diagnosis was correct and challenge it*.”^112^
2.5 Data, laboratory, and records access	Access to patient records and laboratory data was critical for safe prescribing and clinical confidence.	Limited access to patient medical records identified as a major implementation barrier (82.1%).^86^ Lack of access to patient medical records cited as a key barrier (70.1%).^106^ “*The logistics aren't in place for pharmacists to order bloodwork right now… there's no real way for us to monitor safety and efficacy of PrEP*.”^46^
2.6 Information technology systems and platforms	Interoperable, decision-support-enabled IT systems facilitated prescribing, while fragmented platforms hindered implementation.	“*Choose Pharmacy is a fantastic place to store the information but it's not a huge clinical support like the GP systems are…the GP systems will tell you ‘have you checked the renal function’ for example if you're prescribing a nitrofurantoin and they will tell you if there's an interaction if they are penicillin allergic and you try to [prescribe] penicillin it will override it for you*”^80^
2.7 Reimbursement, coverage, and costs	Lack of reimbursement and financial recognition undermined sustainability despite perceived system-level cost savings.	“*Having these prescriptive authorities is great, but if we can't get paid for them you're not gonna see anybody doing it*.”^64^ “…*if pharmacists are not given that provider status to be able to bill insurances for the clinical service of that consultation or taking blood pressure, then pharmacists are not going to do it*.”^52^
2.8 Variation by setting, region and practice type	Prescribing feasibility varied widely by regulatory environment, pharmacy setting, and organisational support.	“I *honestly think especially in rural areas where there are not doctors, or not enough doctors, like where I live right now there are not enough family doctors, that service would probably be used a lot*.”^81^ Pharmacists working in rural or provincial settings were more likely to prefer independent prescribing and minor ailments prescribing compared with their urban counterparts.^95^
**3. Patient-centred access, continuity, and therapeutic relationships**		
3.1 Patient access and convenience	Prescribing improved access to care by reducing wait times and enabling timely treatment, particularly for underserved populations.	The most commonly reported facilitator was accessibility, with more than eight in ten participants (82.8%) noting that ease of access to a pharmacist supported furnishing.^42^ “*You make an appointment to see the physician and it will take you sometimes months to get an appointment… A pharmacist could have helped easily*.”^64^
3.2 Patient satisfaction, trust, and privacy	High patient satisfaction and trust supported uptake, though privacy concerns persisted in some community pharmacy settings.	Satisfaction was extremely high, with 93% stating they were totally satisfied with their visit.^45^ “*We are increasingly being exposed to pharmacist prescribers who seem to make sensible prescribing decisions. I think you're going to feel more confident that if a pharmacist is making a prescribing decision it's going to be a sound one*.”^112^ Concerns about privacy (65.7%).^104^
3.3 Public awareness, marketing and communication	Limited public awareness limited utilisation, highlighting the need for active promotion and clear communication.	“*It's just kind of getting the word out there… different resources to promote these types of services would be really important*.”^46^ One of the most common challenges reported by the pharmacists interviewed was a lack of community awareness of the availability of pharmacist-prescribed contraceptives.^48^
**4. Moral responsibility, risk and safe practice boundaries**		
4.1 Liability and accountability	Concerns about legal responsibility and accountability were prominent barriers, particularly for independent prescribing roles.	“*I think pharmacists are completely unaware of the responsibility they are actually taking on when they start prescribing*.”^112^ “*Even something fairly straightforward such as adjusting a diuretic, you want to be able to follow up and do electrolytes on it, and if you can't and you take legal responsibility for that prescription and something goes wrong, your hands are really tied*”^68^ “*I don't feel comfortable doing it myself because I honestly fear the liability of doing it*.”^64^
4.2 Safety and risk considerations	Patient safety considerations shaped cautious prescribing practices, with preference for protocols, referral, and defined scopes.	“*We are very good at following the rules… if we have a protocol for PrEP prescribing, we would be very good at following that.*”^46^ “*Usually I tend not to go more than a month. That is just my personal safety net as far as prescribing*.”^62^ “*Yeah, the concern about pharmacist prescribing will arise basically from the fact that the competence to be able to make accurate diagnosis is critical in therapy*.”^41^
4.3 Commercial pressures and business model constraints	Tensions between clinical care and retail business models raised concerns about conflicts of interest, despite pharmacists' strong patient-centred professional values.	“*If pharmacists want to become more integrated into the primary healthcare team, I think they are going to have to give up their small shopkeeper empire if you like, and become more integrated and more involved in health centres*”^67^ “*Pharmacists feel that they're under pressure quite a lot and will do things that they know that they shouldn't but feel like they are put under that pressure by their employers*.”^112^ Conflict with pharmacists' business interests may harm the patient.^63^ “… *The one concern I would have is that the person prescribing the drug is also the one profiting from its sale, which could affect which drug is selected by an unscrupulous pharmacist.*”^70^
**5. Professional identity and interprofessional legitimacy**		
5.1 Professional autonomy and role boundaries	Prescribing was commonly viewed as enhancing professional autonomy and clinical legitimacy, though perceptions varied by experience, scope, and role expectations.	“*So, in the end, we're getting away from the principle that we've always fought against, of being drug sellers. And that gives us a boost in terms of being recognised as a healthcare provider. A very good idea.*”^37^ “*Before we had prescribing, we did continuity as well. We just provided the medication and got the ok from the doctor the next day – kind of thing. It just changed the procedure, it hasn't really changed what we do*”^62^ “*If this new role were to take place, I would then feel like my pharmacist meant more to me than just a person counting pills*.”^55^ “*I find it a lot easier to use the GP's prescription pad when writing prescriptions and getting him to sign it and therefore did not have to develop individual CMPs!*”^57^
5.2 Interprofessional dynamics and GP-pharmacy relationships	Relationships with general practitioners strongly influenced acceptance and implementation of prescribing, with collaboration facilitating uptake and resistance constraining it.	“*Peer support and networking cultivates doing this together, so the GPs come to understand what the pharmacist's skills and competencies are. Similarly, that the pharmacist understands the GP, and you start to grow that together and network along with the nurses and the paramedics. So that multidisciplinary approach is going to be really, really important*.”^112^ Some relationships in community practice were less well-established, and in one case, had only been established to facilitate mentoring. Despite this, it was suggested that supplementary prescribing could improve these relationships by promoting better communications.^76^ “*The local doctors have been pretty happy with us looking after their pain management patients. Doctors like it, they feel like we're watching out for our patients*.”^43^ General practitioners located near a pharmacy considered prescribing by referral to be more appropriate than those situated further away, a difference that was statistically significant (*p* = 0.04).^109^

Abbreviations: GP, General Practitioner; PrEP, Pre-Exposure Prophylaxis; IT, Information Technology; CMPs, Clinical Management Plans.

#### 3.3.3 Support, motivation, and clinical readiness

This theme relates to levels of support, pharmacists' motivation, and perceived clinical readiness to engage in prescribing, incorporating perspectives from multiple stakeholders.

##### 3.3.3.1 Support and perceived clinical appropriateness

High levels of support for expanding pharmacist prescribing were reported across pharmacists, patients, and the public.^44^, ^73^, ^82^ Support for pharmacist prescribing was greater when prescribing was embedded within collaborative models of care and supported by multidisciplinary teams and professional support structures.^112^

Support varied by clinical scope. Pharmacists showed greater support for limited or protocol-based antibiotic prescribing in some studies,^39^, ^82^ while support for independent or unrestricted prescribing was considerably lower.^107^ Support for pharmacist prescribing was strongest for clearly defined low-risk conditions such as uncomplicated urinary tract infections.^55^ In vignette-based studies, pharmacists demonstrated high confidence and guideline-concordant prescribing for these presentations, whereas confidence was lower for more complex conditions such as diabetic foot infections,^108^ with such cases also more likely to be referred.^102^ Consistent with this pattern, support dropped markedly for complex or high-risk conditions, including long-term anticoagulation^113^ and prescribing new or complex medications without protocols or physician involvement.^65^, ^74^, ^104^

##### 3.3.3.2 Motivations, professional aspirations, and role perceptions

Prescribing was commonly reported as motivating pharmacists by enabling greater use of clinical training and engagement in patient-centred care,^58^, ^82^ with many also identifying professional development, enhanced professional recognition, and improved professional image as key motivators for expanding professional roles.^39^, ^110^

Patient demand was also an important driver of motivation, particularly in chronic disease management and addressing barriers to timely healthcare access.^51^, ^80^, ^83^ Ethical and public health considerations were also reported as important motivators. This was particularly evident in services such as pre-exposure prophylaxis (PrEP)^46^ and chronic therapy continuation,^44^ which were commonly framed as part of professional responsibility.^86^

Lower motivation was reported among pharmacists working in dispensing-focused or non-clinical roles, where opportunities to apply clinical skills were more limited,^78^ while hesitation related to fear, uncertainty, and perceived scrutiny during early implementation reduced motivation to engage in prescribing for others.^112^ While just over half of pharmacists in one study viewed prescribing as a professional duty, a substantial minority did not, reflecting variation in perceived role boundaries and responsibility.^56^

Perceived lack of recognition from employers, limited financial or career incentives, and low professional esteem for pharmacists further reduced motivation for some.^47^, ^67^ Concerns about competition with other healthcare professionals also discouraged engagement in prescribing roles.^91^ Similarly, reduced motivation was reported where pharmacists perceived limited incentives or recognition associated with prescribing roles.^47^ Despite these challenges, most pharmacists reported a willingness to engage in prescribing roles, particularly where appropriate training, professional support, and governance arrangements were in place,^56^, ^96^ and where opportunities existed to participate in pilot programmes or implement new strategies.^87^, ^97^

##### 3.3.3.3 Preparedness and confidence

Pharmacists generally perceived themselves as reasonably well prepared and increasingly confident to undertake prescribing roles,^56^, ^99^ particularly within low-risk or structured prescribing contexts such as minor ailments,^44^, ^95^ contraception,^87^, ^106^ PrEP,^46^ and medication renewal.^111^ Confidence varied by clinical complexity,^108^ training exposure,^75^ and practice context.^87^, ^114^ Pharmacists expressed confidence in their core medication expertise^66^, with widespread agreement that pharmacists are knowledgeable about medications.^77^ Service users also reported high trust and comfort with pharmacist prescribing, supporting perceived readiness from the patient perspective.^44^, ^45^, ^90^, ^114^

Training and experience were key determinants of confidence. Formal training programmes and protocol use improved pharmacists' comfort with prescribing and clinical decision-making, particularly in knowing when to prescribe versus refer.^75^, ^108^ Experiential learning, including GP shadowing and repeated service provision, was consistently associated with greater confidence than university-based education alone.^57^ However, additional training was sometimes associated with lower perceived confidence among recent graduates, reflecting variability in perceived preparedness.^111^

Preparedness and confidence were further strengthened through engagement in accredited training modules and continuing professional development.^95^, ^113^ Advanced practice pharmacists were more frequently engaged in prescribing^97^; however, some pharmacists expressed reservations regarding additional regulatory requirements, such as specialist registration.^82^ More experienced pharmacists reported higher confidence, whereas newer practitioners perceived more barriers.^50^, ^99^

Confidence was variable across prescribing tasks. Pharmacists reported lower perceived preparedness for managing more complex conditions,^78^, ^98^, ^99^ dose or regimen adaptation,^111^ and high-risk clinical scenarios such as diabetic foot infections^108^ or diabetes management during pregnancy.^98^ Confidence in patient assessment skills was consistently lower than confidence in medicines knowledge, indicating a key boundary of perceived readiness.^66^ Newer pharmacists were sometimes described as more willing to engage with prescribing roles, whereas more experienced practitioners expressed greater caution regarding expanded prescribing responsibilities.^78^ Advanced or clinically embedded pharmacists demonstrated higher confidence and readiness, particularly where supportive interprofessional relationships were present.^97^, ^112^

##### 3.3.3.4 Knowledge and skills gaps

Knowledge and skills gaps were consistently reported as barriers to pharmacist prescribing, particularly relating to clinical assessment, diagnosis, and patient monitoring rather than medicines knowledge.^66^, ^82^ While pharmacists' pharmacological expertise was widely recognised, gaps were identified in diagnosing conditions,^40^ conducting physical assessments,^47^ and managing complex or chronic disease.^99^ These limitations contributed to concerns about the ability to undertake independent clinical decision-making.^55^

Training initiatives were associated with improvements in knowledge, with one study reporting statistically significant gains^75^; however, engagement with prescribing responsibilities frequently revealed additional knowledge gaps.^51^, ^96^, ^100^, ^111^ Consequently, many pharmacists reported a need for additional ongoing training, particularly in clinical assessment and diagnostic reasoning.^38^, ^55^, ^60^, ^89^, ^106^

#### 3.3.4 Structural, policy, and organisational conditions shaping feasibility

Across studies, feasibility of pharmacist prescribing was strongly shaped by organisational structures, system infrastructure, and policy context. Structural enablers determined whether prescribing could be integrated into routine practice, while organisational and system-level constraints limited implementation, sustainability, and scale.

##### 3.3.4.1 Workflow and documentation

Workflow integration and documentation processes were identified as key determinants of feasibility.^61^, ^98^ Pharmacists reported difficulty integrating prescribing into dispensing-focused workflows, particularly in high-volume community pharmacies.^46^, ^71^, ^86^

Prescribing was more feasible where it aligned with existing services (e.g. immunisation, appointment-based care) and where procedural and clinical support resources, such as standardised templates, protocols, and decision-support tools, were available.^46^, ^48^, ^105^ In contrast, documentation burden and frequent interruptions limited prescribing uptake and reduced completion of eligible activities.^75^, ^98^ Concerns related to privacy and confidentiality were also reported, particularly in busy community pharmacy environments where consultation space was limited.^88^, ^92^, ^104^, ^113^

##### 3.3.4.2 Time and staffing pressures

Time constraints and staffing limitations were among the most frequently reported barriers across study designs.^51^, ^69^ Pharmacists described existing workloads as already stretched, expressing concern that prescribing responsibilities could increase pressure and compete with other professional duties.^46^, ^47^

Time-consuming documentation requirements and lack of protected clinical time further limited uptake.^62^ Survey studies identified lack of time and insufficient staffing as key barriers to pharmacist prescribing services across community pharmacy settings.^37^, ^50^ Greater willingness to prescribe was observed among pharmacists who reported adequate staffing and supportive practice conditions.^37^, ^87^

##### 3.3.4.3 Team communication and coordination

Effective communication within pharmacy teams and with other healthcare professionals facilitated implementation, while fragmented communication hindered it.^44^, ^54^, ^85^ Pharmacists embedded within primary care teams reported stronger collaboration than those operating in stand-alone community pharmacy settings.^78^

Clear role delineation, shared protocols, and bidirectional communication were associated with greater service uptake.^47^, ^52^, ^54^ GPs expressed concerns when prescribing occurred without coordination, particularly for more complex therapies.^54^, ^63^

##### 3.3.4.4 Clinical criteria, eligibility, and complexity

Prescribing was more frequently implemented for clearly defined, low-risk conditions guided by protocols,^51^, ^108^ whereas complex cases involving diagnostic uncertainty, multimorbidity, or high-risk therapies were less frequently undertaken by pharmacists in practice.^78^, ^98^ Independent diagnosis was less commonly reported, with pharmacist prescribing more frequently occurring within collaborative or continuation models of care that reflected the defined scope of prescribing roles.^66^, ^83^ Clinical complexity limited implementation, with greater reliance on referral pathways and shared decision-making for higher-risk cases.^102^, ^108^

##### 3.3.4.5 Data, laboratory, and records access

Access to patient records and laboratory data was an important enabler of appropriate and effective prescribing.^39^, ^46^, ^86^, ^104^ Pharmacists with access to electronic medical records reported greater perceived feasibility of prescribing implementation and improved decision support.^80^, ^112^ Lack of access was among the most cited barriers to prescribing, with concerns raised by both pharmacists and GPs regarding information gaps and continuity of care.^39^, ^86^, ^106^

Partial access arrangements enabled documentation and monitoring of prescribing activity,^42^, ^51^ but limitations in access to clinical information constrained fully independent prescribing decisions.^80^, ^106^

##### 3.3.4.6 Information technology systems and platforms

Information technology (IT) infrastructure was reported as both an enabler and a constraint on prescribing implementation.^37^, ^38^ Integrated electronic systems and structured templates supported workflow integration and service delivery,^48^ while limited or poorly integrated systems increased administrative workload.^57^ Many pharmacists reported that existing pharmacy systems lacked clinical functionality available in GP systems, limiting implementation despite professional willingness to prescribe.^57^, ^80^

##### 3.3.4.7 Reimbursement, coverage, and costs

Financial considerations were consistently identified as barriers to implementation.^42^, ^51^, ^69^ Lack of reimbursement, insurance coverage, and provider recognition reduced willingness to prescribe and undermined service sustainability.^52^, ^64^, ^94^ Pharmacists reported difficulty charging consultation fees and absorbing additional workload without compensation.^48^, ^58^, ^113^ Pharmacist prescribing was viewed as potentially cost-saving for the wider health system,^53^ but pharmacies frequently bore the additional workload without corresponding financial remuneration.^38^, ^64^, ^78^

##### 3.3.4.8 Variation by setting, region, and practice type

Feasibility varied by regulatory environment, practice setting, and professional experience.^37^, ^69^, ^95^, ^106^ Prescribing was more commonly reported in jurisdictions with supportive legislation, funding, and established protocols.^56^, ^65^ Independent and rural pharmacies often reported greater confidence and perceived need, whereas larger community pharmacy settings, including chain pharmacies, more frequently cited organisational and workflow constraints.^50^, ^76^, ^84^, ^93^

#### 3.3.5 Patient-centred access, continuity, and therapeutic relationship

This theme captures how pharmacist prescribing was perceived to influence patient access to care, continuity of treatment, and the pharmacist–patient therapeutic relationship. Across the included studies, evidence consistently indicated patient-centred benefits, alongside concerns related to awareness, privacy, and integration with broader healthcare pathways.

##### 3.3.5.1 Patient access and convenience

Improved access and convenience were consistently reported benefits of pharmacist prescribing.^42^, ^79^, ^90^, ^105^ Studies described enhanced access through extended pharmacy opening hours, reduced appointment wait times, and avoidance of physician visits for routine or minor conditions.^44^, ^45^, ^67^, ^93^ Pharmacists reported that prescribing enabled timely initiation or continuation of therapy, particularly for underserved populations.^46^, ^70^, ^83^, ^102^ Survey findings supported these results, with patients and pharmacists frequently reporting increased convenience and perceived improvements in access to care.^65^, ^81^, ^87^

These access benefits were observed across diverse settings, with variation in implementation and experience depending on region and service model.^72^, ^73^, ^94^

In addition to observed benefits, both pharmacists and patients anticipated further improvements in access to care,^65^ especially in rural or underserved areas,^46^, ^101^ alongside reduced pressure on physicians^41^ and direct benefits for patients.^110^ Findings suggest that some patients were willing to pay for pharmacist prescribing services,^81^, ^93^ and pharmacists anticipated that patients would be receptive to expanded prescribing roles.^82^

##### 3.3.5.2 Patient satisfaction, trust, and privacy

Patient satisfaction and trust in pharmacists were commonly reported across included studies.^45^, ^65^, ^79^ Patients valued pharmacists' accessibility, communication style, and perceived expertise, which contributed to high levels of trust and acceptance of prescribing services.^45^, ^112^ Pharmacists also reported favourable patient feedback and willingness to reuse prescribing services.^71^, ^80^, ^93^

##### 3.3.5.3 Public awareness, marketing, and communication

Public awareness of pharmacist prescribing services emerged as a key factor influencing uptake.^46^, ^48^, ^94^ Limited patient awareness reduced utilisation, even in jurisdictions where prescribing services were available.^48^, ^58^ Pharmacists described reliance on passive communication strategies, such as in-pharmacy signage or word of mouth.^42^, ^105^ Low baseline awareness among patients and the general public was commonly reported, with increased acceptance observed following exposure to information about the service.^59^, ^97^ Overall, active communication and targeted promotion were identified as necessary to realise patient-centred benefits at scale.^46^, ^49^

#### 3.3.6 Moral responsibility, risk, and safe practice boundaries

This theme captures how professional responsibility, patient safety, and acceptable boundaries of prescribing practice were conceptualised across stakeholder groups. Evidence from the included studies indicated that concerns related to risk, accountability, and commercial context shaped how the limits of prescribing authority were defined and how the scope of professional responsibility was negotiated.

##### 3.3.6.1 Liability and accountability

Liability and accountability were identified as important considerations across studies.^66^, ^87^, ^110^, ^112^ Several studies reported pharmacists' concerns about legal responsibility for prescribing decisions, particularly where accountability, regulatory clarity, or shared responsibility with other healthcare professionals was uncertain.^64^, ^68^, ^98^, ^112^

Pharmacists in qualitative studies expressed uncertainty regarding professional indemnity coverage, responsibility for adverse outcomes, and medico-legal risk when prescribing independently.^64^, ^78^ Survey studies supported these findings, with a substantial proportion of respondents identifying liability concerns as a barrier to participation in prescribing services.^75^, ^110^

##### 3.3.6.2 Safety and risk considerations

Patient safety was consistently framed as a central professional and ethical consideration influencing prescribing decisions.^76^, ^85^, ^92^, ^106^ Pharmacists emphasised cautious patient selection, adherence to protocols, and awareness of personal competence limits, with many preferring to practise within clearly defined scopes and structured governance frameworks.^46^, ^62^, ^76^, ^112^ Pharmacists described managing risk through conservative decision-making, referral to other providers when appropriate, and adherence to established clinical guidelines or structured protocols.^46^, ^102^, ^112^

Elevated levels of concern were reported regarding safety, particularly in relation to diagnostic uncertainty and patient complexity, with limited access to health records further amplifying perceived risk.^66^, ^78^, ^113^ Safety concerns were more pronounced in studies examining broader or less protocol-driven prescribing roles, where independent diagnosis was perceived as carrying greater risk.^76^, ^112^

##### 3.3.6.3 Commercial pressures and business model constraints

The tension between clinical responsibility and the commercial nature of pharmacy practice was identified as an ethical issue in some studies.^63^, ^68^ Pharmacists, patients, and other healthcare professionals expressed apprehension that prescribing within a retail environment could create real or perceived conflicts of interest, particularly where prescribers also dispense the medicines they prescribe.^55^, ^66^, ^70^

Concerns were raised that commercial incentives and the dual role of prescribing and dispensing might influence prescribing decisions or undermine public trust in pharmacists' professional judgement.^55^, ^67^, ^70^ At the same time, several studies highlighted pharmacists' strong professional identity and intrinsic motivation to prioritise patient care, with pharmacists challenging perceptions that prescribing would be driven by financial gain.^67^, ^78^ Prescribing was framed by pharmacists as an extension of professional responsibility and patient advocacy rather than a commercial opportunity,^63^, ^78^ although concerns about public perception and the optics of retail-based prescribing remained.^63^, ^66^

#### 3.3.7 Professional identity, autonomy, and legitimacy

Pharmacist prescribing was closely linked to professional identity, autonomy, and perceived legitimacy within the wider healthcare system. Across studies, prescribing was associated with how pharmacists understood their clinical role and how that role was recognised by other healthcare professionals and the public.

##### 3.3.7.1 Professional identity and perceived autonomy

Prescribing was frequently described as a marker of professional advancement and clinical legitimacy, with many pharmacists reporting that it enabled more complete use of their clinical training and supported movement beyond a dispensing-focused role.^37^, ^48^, ^58^ For many, prescribing was framed as legitimising activities already embedded in routine practice, including therapy continuation and informal adaptations to medicines or prescriptions, rather than representing a fundamental change in role.^62^, ^78^

Prescribing was commonly described as an expansion of pharmacists’ clinical roles, contributing to more positive perceptions of pharmacists' professional duties.^58^, ^74^, ^104^, ^114^ However, variation was reported in how pharmacists incorporated prescribing into their professional identity and perceived level of autonomy. Some expressed uncertainty or preferred prescribing to remain protocol-driven or undertaken within collaborative or supervised models, particularly in areas such as antibiotic use or broader scope expansion.^39^, ^44^, ^57^

Concerns about role confusion and boundary overlap with medical practitioners were reported by pharmacists, physicians, and the public, including uncertainty regarding clinical responsibility and decision-making authority.^54^, ^66^, ^82^, ^85^

Perceptions of prescribing as part of professional identity varied across pharmacists, with differences linked to professional experience and interpretations of pharmacists' clinical role. Some pharmacists described prescribing as aligning with an expanded clinical identity, while others reported caution linked to traditional role expectations and the increased responsibility associated with prescribing practice.^41^, ^78^, ^112^

##### 3.3.7.2 Interprofessional dynamics and GP-pharmacy relationships

Interprofessional relationships, particularly with general practitioners (GPs), were identified as an important influence on the legitimacy and implementation of pharmacist prescribing.^56^, ^76^, ^78^ Supportive GP-pharmacist relationships characterised by collaboration, trust, and clear communication pathways were associated with greater legitimacy and acceptance of prescribing roles, particularly within collaborative or continuation-of-care models.^43^, ^44^, ^55^, ^76^ In contrast, resistance from physicians, concerns about role encroachment, and lack of shared understanding of prescribing boundaries were reported as barriers to implementation.^41^, ^76^, ^109^ Perceptions of physician support were mixed, with acceptance often conditional on scope limitations, GP oversight, or referral-based models.^40^, ^103^, ^104^

Overall, interprofessional dynamics varied across jurisdictions, practice settings, and regulatory frameworks, reflecting differences in how prescribing authority, oversight, and professional boundaries were negotiated between pharmacists and medical practitioners and shaping both the uptake and sustainability of pharmacist prescribing services.^41^, ^52^, ^71^, ^109^

### 3.4 Implementation stage analysis

The distribution of coded excerpts across implementation stages is presented in Appendix 6. A similar volume of data was available for pre-implementation (*n* = 638) and post-implementation (*n* = 673) stages, with comparatively fewer excerpts derived from peri-implementation contexts (*n* = 98), reflecting the smaller number of studies explicitly examining the implementation phase itself. Analysis showed that all five analytical themes were present across pre-, peri- and post-implementation contexts, indicating that similar stakeholder considerations were reported throughout the implementation process. However, the prominence of excerpts varied across stages. Codes relating to structural, policy and organisational conditions shaping feasibility were more frequently reported in post-implementation studies (*n* = 169) compared with pre-implementation (*n* = 90). Excerpts relating to support, motivation, and clinical readiness were highly represented in both pre-implementation (*n* = 281) and post-implementation (*n* = 250) contexts. Excerpts relating to patient-centred access, continuity and therapeutic relationship, moral responsibility, risk and safe practice boundaries, and professional identity, autonomy and legitimacy were distributed relatively evenly across implementation stages. At the level of descriptive subthemes, no consistent or systematic differences were identified between stages.

## 4 Discussion

This review broadly found support for pharmacist prescribing in community pharmacy settings across stakeholder groups (including patients, pharmacists, and other healthcare professionals), although views varied across groups and contexts.^44^, ^73^, ^82^ The review also showed that this prescribing is not determined by individual competence alone, but by the extent to which it is supported within organisational and system-level structures. Implementation was not uniform, with variation in uptake associated with differences in training, confidence, patient acceptance, and organisational and system-level support.^75^, ^77^, ^99^, ^112^ Therefore, this emphasises that stakeholder acceptance alone does not necessarily ensure uptake. Instead, the findings suggest that prescribing is shaped by how well it is supported within existing clinical systems, service structures, and policy environments. Pharmacist prescribing should therefore be implemented as part of a coordinated, system-level strategy rather than relying solely on individual capability or training. This interpretation is in line with findings from previous literature reviews, which emphasise that the implementation of non-medical prescribing is influenced by interacting individual, organisational, and system-level determinants rather than isolated professional competence.^3^, ^4^, ^18^, ^20^ It also aligns with implementation research highlighting the importance of multi-level determinants in shaping practice change.^21^, ^22^

Pharmacist prescribing is a developing practice with increasing research focus, indicated by more than half of this review's included studies being published between 2020 and 2025, reflecting both the rapid expansion of prescribing practice^4^, ^12^, ^13^ and growing research in this field. The present systematic review extends the evidence from previous reviews^6^, ^7^, ^9^, ^18^, ^19^, ^20^ by focusing specifically on community pharmacy and bringing together the perspectives of multiple stakeholder groups across different prescribing models and stages of implementation. Rather than focusing on one stakeholder type, one prescribing model, or identifying barriers and facilitators in isolation, this review's thematic synthesis more deeply describes how individual organisational and system-level factors interact to shape implementation. It provides a more detailed understanding of why stakeholder support for pharmacist prescribing does not necessarily translate into consistent implementation into routine practice. Therefore, this review may be particularly useful to future researchers when implementing pharmacist prescribing, as the present findings can be simply mapped to an established implementation science framework to suit their needs in different settings.

A key finding across the included studies is that prescribing capability is highly context dependent. Confidence and readiness were greater in structured, protocol-driven contexts but reduced in more complex situations requiring diagnostic reasoning.^87^, ^98^, ^99^, ^108^ This suggests that prescribing capability varies according to clinical context and available support with clear implications for workforce development. Training and support structures should prioritise diagnostic reasoning and clinical decision-making in complex situations, rather than focusing predominantly on protocol-based prescribing. This reflects international competency frameworks, including those developed by the International Pharmaceutical Federation (FIP), which emphasise clinical reasoning, patient assessment, and reflective practice as core elements of advanced pharmacy practice, aspects that are directly relevant to prescribing roles.^115^ Similar trends are reported in non-medical prescribing literature, where confidence develops through experience, structured training, and supported practice environments,^3^, ^18^, ^19^ and where education, supervision, and practice-based learning are central to safe prescribing practice.^7^, ^20^ Expanding prescribing safely will depend on strengthening clinical decision-making capabilities, not just extending prescribing authority.

Risk is closely linked to this context dependence and shapes prescribing boundaries. Rather than acting solely as a barrier, it functions as a regulatory mechanism. Concerns related to liability and professional accountability reinforce cautious approaches and limit engagement in perceived higher-risk or uncertain scenarios.^64^, ^68^, ^75^, ^110^ This is consistent with broader non-medical prescribing literature, highlighting increased professional responsibility, accountability, and exposure to clinical risk.^3^, ^18^, ^20^ These findings emphasise the need for clear governance frameworks, clinical support systems, and supervision structures to enable safe expansion of prescribing practice.

Organisational conditions further shape how prescribing is enacted. Implementation is facilitated where prescribing can be integrated into existing workflows and supported by appropriate systems, and resources,^48^, ^61^, ^80^ but constrained where workload pressures, staffing limitations, and system fragmentation persist.^47^, ^51^, ^61^, ^86^ These findings align with literature identifying organisational readiness, workload capacity, and access to clinical information as key determinants of implementation^7^, ^18^, ^19^, ^20^, ^21^, ^23^, reinforcing the need for organisational redesign, including integration into existing workflows and access to clinical infrastructure. Policy direction in the UK and Ireland reflects this approach, where prescribing is being developed through structured service models, funding frameworks, and defined implementation pathways.^116^, ^117^ Without these structural supports, prescribing is unlikely to be sustained at scale. The importance of these factors may also differ between healthcare systems as prescribing models and implementation are influenced by regulatory, organisational and financial contexts.^3^, ^18^, ^20^ For example, concerns about reimbursement, commercial interests and medico-legal responsibility may vary depending on how healthcare is funded and organised.^3^, ^18^ The review did not formally compare findings by healthcare system type, so differences between systems cannot be determined from this synthesis. Most studies were also conducted in high-income countries, with limited evidence from low- and middle-income jurisdictions. The findings may therefore not fully reflect the challenges of implementing pharmacist prescribing in different healthcare settings. Evidence from low- and middle-income countries and other underrepresented settings is needed to understand how implementation varies across healthcare systems. Future researchers could also explore how healthcare funding, regulation, and the role of community pharmacy within the healthcare system influence implementation.

Prescribing is also shaped by professional legitimacy and interprofessional relationships. Engagement is greater where roles are clearly defined and aligned with existing responsibilities, and more limited where uncertainty exists regarding scope or overlap with medical roles.^41^, ^76^, ^78^, ^109^ This reflects wider literature indicating that extended clinical roles depend on negotiated boundaries and interprofessional trust.^6^, ^8^, ^18^, ^20^ Implementation strategies should therefore actively support interprofessional collaboration and role clarity, as trust and shared understanding are critical to the successful integration of prescribing into practice. Professional acceptance alone is insufficient without clear role definition and collaborative practice structures.

While findings largely align with existing literature,^7^, ^9^, ^18^, ^19^, ^20^ several tensions were identified. Although confidence is often assumed to increase with experience, this review indicates that it remains context dependent, particularly in complex or uncertain situations.^87^, ^98^, ^99^ Similarly, while prescribing is associated with improved access and outcomes, these benefits are not inherent but depend on how services are organised and integrated.^6^, ^16^, ^61^ Prescribing is often framed as an extension of professional autonomy; however, in practice it was commonly enacted within structured, protocol-driven, or collaborative models, suggesting autonomy is moderated by risk and organisational constraints.^76^, ^78^, ^109^ Finally, despite being described as established, implementation remains uneven, with persistent organisational and interprofessional barriers limiting full integration into routine care.^41^, ^51^, ^78^, ^86^ These tensions indicate that pharmacist prescribing is more conditional and context-sensitive than often assumed, and has important implications for how prescribing translates into patient outcomes. Improvements in access, convenience, and patient satisfaction and trust were reported,^42^, ^45^, ^79^, ^90^ particularly where prescribing services were easily accessible and integrated into care pathways,^46^, ^79^, ^105^ but were reduced where services lacked visibility, coordination, or resources.^48^, ^70^, ^88^, ^92^ This is consistent with wider evidence highlighting the importance of service integration and accessibility in supporting patient-centred outcomes.^2^, ^9^, ^19^ Improving patient outcomes requires not only expanding prescribing roles, but also ensuring that services are visible, accessible, and fully integrated within the wider healthcare system.

Several priorities emerge for policy, practice, and research. Implementation should prioritise system-level readiness, including workforce capacity, access to clinical information, and integration within existing care pathways.^48^, ^61^, ^80^, ^86^ Prescribing roles need to be embedded within clearly defined service models, supported by governance frameworks, supervision, and interprofessional collaboration.^61^, ^76^, ^78^, ^109^ Training should extend beyond protocol-based prescribing to include diagnostic reasoning and management of more complex cases, reflecting the context-dependent nature of prescribing capability identified in this review^78^, ^98^, ^99^. In parallel, further research is needed to address key gaps, including prescribing in more complex clinical contexts, how services are sustained over time (e.g. longitudinal implementation studies), and the extent to which prescribing improves patient and system-level outcomes such as safety, effectiveness, and cost-effectiveness (e.g. through economic evaluations).^16^, ^44^, ^61^, ^73^ This review's findings also highlight the need for further research to examine the role of digital systems and shared clinical records in supporting implementation. Further work is also needed to understand how interprofessional relationships and role boundaries evolve as prescribing becomes more embedded.^41^, ^76^, ^78^, ^109^ Progress on these fronts will determine whether prescribing becomes a routine feature of community pharmacy practice or remains unevenly embedded.

Overall, implementation depends on alignment across individual, organisational, and system levels, and limitations at any level can limit uptake and sustainability. Without this alignment, prescribing is likely to remain dependent on local conditions rather than functioning as a consistent, system-wide service. This highlights the need to approach pharmacist prescribing as a system-wide change, requiring coordinated action across education, workforce planning, service design, and policy.

### 4.1 Strengths and limitations

A key strength of this review is the use of a convergent integrated mixed-methods approach,^26^ enabling inclusion of a large and diverse evidence base (*n* = 78) and integration of quantitative trends with qualitative insights. The inclusion of multiple stakeholder groups and diverse study designs further supports a comprehensive understanding of factors influencing the implementation of pharmacist prescribing. However, variation in study designs, stakeholder groups, healthcare systems, prescribing models, and regulatory contexts may have somewhat limited comparability, and thus may limit the transferability (e.g. beyond high-income settings) and overall generalisability of some findings. Additionally, many studies relied on convenience sampling and self-reported data, further limiting generalisability. Restricting inclusion to English-language, published studies may also have introduced language and publication bias.

Lastly, the use of thematic synthesis^34^ strengthens the findings by generating higher-level interpretive insights and is particularly useful in communicating complex evidence to a wide variety of stakeholders. The sensitivity analysis further strengthened the robustness of the findings, confirming that the main themes remained consistent following exclusion of studies with greater methodological limitations.

## 5 Conclusion

Pharmacist prescribing in the community pharmacy setting is a system-dependent practice, shaped less by individual competence than by the organisational and policy conditions in which it operates. Where workflow integration, access to clinical information, governance frameworks, and interprofessional collaboration are in place, prescribing can be more effectively integrated into routine practice. Where these conditions are absent, uptake may be limited and prescribing more difficult to sustain. Expanding prescribing therefore requires coordinated action across policy, workforce development, and service design; without this alignment, uptake is likely to remain uneven. These findings offer clear direction for policymakers, regulators, and healthcare organisations on the structural conditions necessary for pharmacist prescribing to function as a routine, integrated component of primary care, rather than an adjunct dependent on individual initiative.

## CRediT authorship contribution statement

**Anna Colthorpe:** Writing – review & editing, Writing – original draft, Visualization, Validation, Software, Project administration, Methodology, Investigation, Formal analysis, Data curation, Conceptualization. **Aoife Fleming:** Writing – review & editing, Visualization, Supervision, Methodology, Funding acquisition, Conceptualization. **Suzanne McCarthy:** Writing – review & editing, Visualization, Supervision, Methodology, Funding acquisition, Conceptualization. **Eimear McManus:** Validation, Investigation, Data curation. **John J Keating:** Writing – review & editing, Visualization, Supervision, Methodology, Funding acquisition, Conceptualization. **Kieran Dalton:** Writing – review & editing, Writing – original draft, Validation, Supervision, Methodology, Funding acquisition, Conceptualization.

## Funding

This research received no external funding; it was conducted as part of Anna Colthorpe's PhD, funded by the School of Pharmacy, University College Cork.

## Declaration of competing interest

The authors declare that they have no known competing financial interests or personal relationships that could have appeared to influence the work reported in this paper.
