# Supplementary material for: Stakeholders' views of the implementation of pharmacist prescribing in the community pharmacy setting: a mixed-methods systematic review
Source: Explor Res Clin Soc Pharm. 2026 Sep 19;24:100846. doi: 10.1016/j.rcsop.2026.100846 (PMC13639159; doi:10.1016/j.rcsop.2026.100846)
Supplement: Supplementary file 1 [file mmc1.docx]

**Appendices**

Appendix 1: PRISMA 2020 Checklist

Appendix 2: PRISMA-S Checklist

Appendix 3: Search Strategy

Appendix 4: Contributions of Studies to the Synthesis

Appendix 5: Quality Assessment with Mixed Methods Appraisal Tool (MMAT)

Appendix 6: Distribution of Analytical Theme codes across Implementation Stages

**Note**: This appendices document accompanies the main article titled ‘*Stakeholders' views of the implementation of pharmacist prescribing in the community pharmacy setting: a mixed-methods systematic review’*.

**Appendix 1: PRISMA 2020 Checklist**

| **Section and Topic** | **Item #** | **Checklist item** | **Location where item is reported** |
| --- | --- | --- | --- |
| **TITLE** | | | |
| Title | 1 | Identify the report as a systematic review. | Title |
| **ABSTRACT** | | | |
| Abstract | 2 | See the PRISMA 2020 for Abstracts checklist. | Abstract |
| **INTRODUCTION** | | | |
| Rationale | 3 | Describe the rationale for the review in the context of existing knowledge. | Introduction |
| Objectives | 4 | Provide an explicit statement of the objective(s) or question(s) the review addresses. | Introduction |
| **METHODS** | | | |
| Eligibility criteria | 5 | Specify the inclusion and exclusion criteria for the review and how studies were grouped for the syntheses. | Methods (Eligibility criteria) |
| Information sources | 6 | Specify all databases, registers, websites, organisations, reference lists and other sources searched or consulted to identify studies. Specify the date when each source was last searched or consulted. | Methods (Search strategy); Appendix 3 |
| Search strategy | 7 | Present the full search strategies for all databases, registers and websites, including any filters and limits used. | Appendix 3 |
| Selection process | 8 | Specify the methods used to decide whether a study met the inclusion criteria of the review, including how many reviewers screened each record and each report retrieved, whether they worked independently, and if applicable, details of automation tools used in the process. | Methods (Study selection) |
| Data collection process | 9 | Specify the methods used to collect data from reports, including how many reviewers collected data from each report, whether they worked independently, any processes for obtaining or confirming data from study investigators, and if applicable, details of automation tools used in the process. | Methods (Data extraction) |
| Data items | 10a | List and define all outcomes for which data were sought. Specify whether all results that were compatible with each outcome domain in each study were sought (e.g. for all measures, time points, analyses), and if not, the methods used to decide which results to collect. | Methods (Data synthesis) |
|  | 10b | List and define all other variables for which data were sought (e.g. participant and intervention characteristics, funding sources). Describe any assumptions made about any missing or unclear information. | Methods (Data extraction) |
| Study risk of bias assessment | 11 | Specify the methods used to assess risk of bias in the included studies, including details of the tool(s) used, how many reviewers assessed each study and whether they worked independently, and if applicable, details of automation tools used in the process. | Methods (Quality Assessment); Appendix 5 |
| Effect measures | 12 | Specify for each outcome the effect measure(s) (e.g. risk ratio, mean difference) used in the synthesis or presentation of results. | Not applicable |
| Synthesis methods | 13a | Describe the processes used to decide which studies were eligible for each synthesis (e.g. tabulating the study intervention characteristics and comparing against the planned groups for each synthesis (item #5)). | Methods (Data extraction, Data synthesis) |
|  | 13b | Describe any methods required to prepare the data for presentation or synthesis, such as handling of missing summary statistics, or data conversions. | Methods (Data extraction, Data synthesis) |
|  | 13c | Describe any methods used to tabulate or visually display results of individual studies and syntheses. | Methods (Data extraction, Data synthesis) |
|  | 13d | Describe any methods used to synthesize results and provide a rationale for the choice(s). If meta-analysis was performed, describe the model(s), method(s) to identify the presence and extent of statistical heterogeneity, and software package(s) used. | Methods (Data synthesis) |
|  | 13e | Describe any methods used to explore possible causes of heterogeneity among study results (e.g. subgroup analysis, meta-regression). | Methods (Data synthesis) |
|  | 13f | Describe any sensitivity analyses conducted to assess robustness of the synthesized results. | Methods (Quality assessment) |
| Reporting bias assessment | 14 | Describe any methods used to assess risk of bias due to missing results in a synthesis (arising from reporting biases). | Methods (Search strategy, Rigour and reflexivity) |
| Certainty assessment | 15 | Describe any methods used to assess certainty (or confidence) in the body of evidence for an outcome. | Not applicable |
| **RESULTS** | | | |
| Study selection | 16a | Describe the results of the search and selection process, from the number of records identified in the search to the number of studies included in the review, ideally using a flow diagram. | Results (Search results,  Figure 1) |
|  | 16b | Cite studies that might appear to meet the inclusion criteria, but which were excluded, and explain why they were excluded. | Results (Search results,  Figure 1) |
| Study characteristics | 17 | Cite each included study and present its characteristics. | Results (Characteristics of included studies, Tables 1 and 2) |
| Risk of bias in studies | 18 | Present assessments of risk of bias for each included study. | Appendix 5 |
| Results of individual studies | 19 | For all outcomes, present, for each study: (a) summary statistics for each group (where appropriate) and (b) an effect estimate and its precision (e.g. confidence/credible interval), ideally using structured tables or plots. | Not applicable |
| Results of syntheses | 20a | For each synthesis, briefly summarise the characteristics and risk of bias among contributing studies. | Results (Characteristics of included studies, Quality of included studies, Descriptive and analytical themes) |
|  | 20b | Present results of all statistical syntheses conducted. If meta-analysis was done, present for each the summary estimate and its precision (e.g. confidence/credible interval) and measures of statistical heterogeneity. If comparing groups, describe the direction of the effect. | Not applicable |
|  | 20c | Present results of all investigations of possible causes of heterogeneity among study results. | Results (Implementation stage analysis); Appendix 6 |
|  | 20d | Present results of all sensitivity analyses conducted to assess the robustness of the synthesized results. | Results (Quality of included studies); Appendix 4 |
| Reporting biases | 21 | Present assessments of risk of bias due to missing results (arising from reporting biases) for each synthesis assessed. | Results (Quality of included studies); Appendix 5 |
| Certainty of evidence | 22 | Present assessments of certainty (or confidence) in the body of evidence for each outcome assessed. | Results (Quality of included studies); Appendix 5 |
| **DISCUSSION** | | | |
| Discussion | 23a | Provide a general interpretation of the results in the context of other evidence. | Discussion |
|  | 23b | Discuss any limitations of the evidence included in the review. | Discussion |
|  | 23c | Discuss any limitations of the review processes used. | Discussion |
|  | 23d | Discuss implications of the results for practice, policy, and future research. | Discussion |
| **OTHER INFORMATION** | | | |
| Registration and protocol | 24a | Provide registration information for the review, including register name and registration number, or state that the review was not registered. | Methods (PROSPERO; CRD42025646376) |
|  | 24b | Indicate where the review protocol can be accessed, or state that a protocol was not prepared. | Methods PROSPERO; (https://www.crd.york.ac.uk/PROSPERO/view/CRD42025646376) |
|  | 24c | Describe and explain any amendments to information provided at registration or in the protocol. | Methods (Search strategy) |
| Support | 25 | Describe sources of financial or non-financial support for the review, and the role of the funders or sponsors in the review. | Funding |
| Competing interests | 26 | Declare any competing interests of review authors. | Conflicts of interest |
| Availability of data, code and other materials | 27 | Report which of the following are publicly available and where they can be found: template data collection forms; data extracted from included studies; data used for all analyses; analytic code; any other materials used in the review. | Not applicable |

*From:*  Page MJ, McKenzie JE, Bossuyt PM, Boutron I, Hoffmann TC, Mulrow CD, et al. The PRISMA 2020 statement: an updated guideline for reporting systematic reviews. BMJ 2021;372:n71. doi: 10.1136/bmj.n71. This work is licensed under CC BY 4.0. To view a copy of this license, visit <https://creativecommons.org/licenses/by/4.0/>

**Appendix 2: PRISMA-S Checklist**

| **Section/topic** | **#** | | **Checklist item** | **Location(s) Reported** |
| --- | --- | --- | --- | --- |
| **INFORMATION SOURCES AND METHODS** | | | | |
| Database name | 1 | | Name each individual database searched, stating the platform for each. | Search Strategy |
| Multi-database searching | 2 | | If databases were searched simultaneously on a single platform, state the name of the platform, listing all of the databases searched. | Not applicable |
| Study registries | 3 | | List any study registries searched. | Not applicable |
| Online resources and browsing | 4 | | Describe any online or print source purposefully searched or browsed (e.g., tables of contents, print conference proceedings, web sites), and how this was done. | Not applicable |
| Citation searching | 5 | | Indicate whether cited references or citing references were examined, and describe any methods used for locating cited/citing references (e.g., browsing reference lists, using a citation index, setting up email alerts for references citing included studies). | Search Strategy |
| Contacts | 6 | | Indicate whether additional studies or data were sought by contacting authors, experts, manufacturers, or others. | Not applicable |
| Other methods | 7 | | Describe any additional information sources or search methods used. | Search Strategy |
| **SEARCH STRATEGIES** | | | | |
| Full search strategies | 8 | | Include the search strategies for each database and information source, copied and pasted exactly as run. | Appendix 3 |
| Limits and restrictions | 9 | | Specify that no limits were used, or describe any limits or restrictions applied to a search (e.g., date or time period, language, study design) and provide justification for their use. | Eligibility Criteria,  Supplementary Material |
| Search filters | 10 | | Indicate whether published search filters were used (as originally designed or modified), and if so, cite the filter(s) used. | Not applicable |
| Prior work | 11 | | Indicate when search strategies from other literature reviews were adapted or reused for a substantive part or all of the search, citing the previous review(s). | Not applicable |
| Updates | 12 | | Report the methods used to update the search(es) (e.g., rerunning searches, email alerts). | Search Strategy |
| Dates of searches | 13 | | For each search strategy, provide the date when the last search occurred. | Search Strategy |
| **PEER REVIEW** | | | | |
| Peer review | 14 | | Describe any search peer review process. | Not applicable |
| **MANAGING RECORDS** | | | | |
| Total Records | 15 | | Document the total number of records identified from each database and other information sources. | Figure 1 PRISMA Flow Diagram |
| Deduplication | 16 | | Describe the processes and any software used to deduplicate records from multiple database searches and other information sources. | Study Selection |
|  |  | |  |  |
| PRISMA-S: An Extension to the PRISMA Statement for Reporting Literature Searches in Systematic Reviews | | | |  |
| Rethlefsen ML, Kirtley S, Waffenschmidt S, Ayala AP, Moher D, Page MJ, Koffel JB, PRISMA-S Group.  Last updated February 27, 2020. | | | |  |
|  | |  |  |  |
|  | |  |  |  |

**Appendix 3: Search Strategy**

All searches were carried out on 3^rd^ March 2025

**1. PubMed (pubmed.gov):**

(((Pharmacist*[Title/Abstract] AND Prescrib*[Title/Abstract]) AND (view* OR experience* OR feeling* OR thought* OR opinion* OR idea* OR belief* OR attitude* OR perspective* OR perception* OR barrier* OR obstacle* OR challenge* OR enabler* OR facilitator*)) AND (qualitative* OR interview* OR focus group* OR survey* OR questionnaire* OR diar*)) AND (Pharmacy or "Primary care" or "Community care")

**2. Embase (Ovid):**

S1: (Pharmacist* NEAR/6 Prescrib*):ab,ti

S2: (view* OR experience* OR feeling* OR thought* OR opinion* OR idea* OR belief* OR attitude* OR perspective* OR perception* OR barrier* OR obstacle* OR challenge* OR enabler* OR facilitator*) AND [humans]/lim

S3: (qualitative* OR interview* OR focus NEXT/1 group* OR survey* OR questionnaire* OR diar*)

S4: (Pharmacy or (Primary NEAR/1 care) or (Community NEAR/1 care)) AND [humans]/lim

S5: S1 AND S2 AND S3 AND S4

**3. CINAHL (EBSCO):**

S1: (Pharmacist* AND Prescrib*) [Abstract]

S2: (view* OR experience* OR feeling* OR thought* OR opinion* OR idea* OR belief* OR attitude* OR perspective* OR perception* OR barrier* OR obstacle* OR challenge* OR enabler* OR facilitator*) [TX All Text]

S3: (qualitative* OR interview* OR focus group* OR survey* OR questionnaire* OR diar*) [TX All Text] AND Pharmacy or Primary care or Community care [TX All Text]

S4: S1 AND S2 AND S3

**4. Scopus (Elsevier):**

S1: ALL (view* OR experience* OR feeling* OR thought* OR opinion* OR idea* OR belief* OR attitude* OR perspective* OR perception*)

S2: ALL (barrier* OR obstacle* OR challenge* OR enabler* OR facilitator*)

S3: ALL (qualitative* OR interview* OR focus group* OR survey* OR questionnaire* OR diar*)

S4: ALL ( pharmacy OR ( primary W/1 care) OR (community W/1 care) )

S5: TITLE-ABS-KEY (pharmacist* W/6 prescrib* )

S6: S1 AND S2 AND S3 AND S4 AND S5

**5. Web of Science Core Collection (Clarivate):**

S1: AB=(prescrib* NEAR/6 pharmacist*)

S2: ALL=(Pharmacy OR "primary care" OR "community care")

S3: ALL=(view* OR experience* OR feeling* OR thought* OR opinion* OR idea* OR belief* OR attitude* OR perspective* OR perception* OR barrier* OR obstacle* OR challenge* OR enabler* OR facilitator*)

S4: ALL=(qualitative* OR interview* OR focus group* OR survey* OR questionnaire* OR diar*)

S5: S1 AND S2 AND S3 AND S4

**Appendix 4:** **Contributions of Studies to the Synthesis**

| **Study** | **Support, Motivation & Clinical Readiness** | **Structural, Policy & Organisational Conditions Shaping Feasibility** | **Patient-Centred Access, Continuity & Therapeutic Relationship** | **Moral Responsibility, Risk & Safe Practice Boundaries** | **Professional Identity, Autonomy & Legitimacy** | **Study Design** |
| --- | --- | --- | --- | --- | --- | --- |
| Amador-Fernández *et al.* 2023 | - | Yes | - | - | Yes | Qualitative |
| Amador-Fernández *et al.* 2024 | Yes | Yes | - | - | - | Mixed Methods |
| AttardPizzuto *et al.* 2019 | Yes | Yes | - | - | Yes | Quantitative |
| Auta *et al.* 2014 | Yes | - | - | - | Yes | Quantitative |
| Auta *et al.* 2016 | Yes | - | Yes | - | Yes | Qualitative |
| Azad *et al.* 2024 | - | Yes | Yes | - | - | Mixed Methods |
| Banawis *et al.* 2023 | - | - | - | - | Yes | Mixed Methods |
| Baratta *et al.* 2025 | Yes | Yes | Yes | - | Yes | Quantitative |
| Beahm *et al.* 2018 | Yes | - | Yes | - | - | Quantitative |
| Booker *et al.* 2023 | Yes | Yes | Yes | Yes | - | Mixed Methods |
| BrilliantMoloto *et al*. 2024 | Yes | Yes | - | - | - | Qualitative |
| Campi *et al.* 2024 | - | Yes | Yes | - | Yes | Qualitative |
| Chuene *et al.* 2024 | - | - | Yes | - | - | Qualitative |
| Cohen *et al.* 2024 | Yes | Yes | - | - | - | Quantitative |
| Dahri *et al.* 2024 | Yes | Yes | - | - | - | Quantitative |
| Dale *et al.* 2024 | - | Yes | - | - | Yes | Qualitative |
| Famiyeh *et al*. 2019 | - | Yes | - | - | - | Qualitative |
| Faruquee *et al.* 2019 | - | Yes | - | - | Yes | Qualitative |
| Feehan *et al*. 2016 | Yes | - | - | Yes | Yes | Qualitative |
| George *et al.* 2006 | Yes | Yes | - | - | Yes | Quantitative |
| George *et al.* 2007 | Yes | Yes | - | - | Yes | Quantitative |
| Gomez *et al.* 2020 | Yes | Yes | Yes | - | Yes | Qualitative |
| Gomez *et al*. 2022 | - | - | Yes | - | - | Quantitative |
| Grant *et al*. 2023 | Yes | - | - | - | - | Mixed Methods |
| Grant *et al.* 2025 | - | Yes | - | - | - | Mixed Methods |
| Guirguis *et al.* 2014 | - | - | - | Yes | Yes | Qualitative |
| Hatah *et al*. 2013 | - | Yes | - | Yes | - | Qualitative |
| Herman et al. 2020 | - | Yes | - | Yes | - | Qualitative |
| Hoti *et al*. 2011 | Yes | Yes | Yes | - | - | Quantitative |
| Hoti *et al*. 2013 | Yes | Yes | - | Yes | Yes | Quantitative |
| Hughes *et al*. 2003 | Yes | - | Yes | Yes | - | Qualitative |
| Hughes *et al*. 2014 | - | - | - | Yes | - | Qualitative |
| Hunter *et al.* 2023 | - | Yes | - | - | - | Quantitative |
| Irwin *et al.* 2019 | - | - | Yes | Yes | - | Mixed Methods |
| Karim *et al*. 2024 | - | Yes | Yes | - | Yes | Quantitative |
| Koester *et al.* 2020 | - | - | Yes | - | - | Qualitative |
| Lemay *et al.* 2025 | Yes | - | Yes | - | - | Quantitative |
| Lewis *et al*. 2021 | Yes | - | - | - | Yes | Quantitative |
| Lio *et al*. 2018 | Yes | Yes | - | Yes | - | Quantitative |
| Lloyd *et al*. 2007 | - | Yes | - | Yes | Yes | Qualitative |
| Lutz *et al.* 2021 | Yes | - | - | - | - | Quantitative |
| Makowsky *et al.* 2013 | Yes | Yes | - | Yes | Yes | Qualitative |
| Mansell *et al.*2015 | - | - | Yes | - | - | Quantitative |
| Mantzourani *et al.* 2023 | Yes | Yes | Yes | - | - | Qualitative |
| Meredith *et al*. 2020 | - | - | Yes | - | - | Qualitative |
| Mhailan *et al.* 2022 | Yes | - | Yes | - | Yes | Quantitative |
| Miszewska *et al.* 2022 | Yes | Yes | Yes | - | - | Quantitative |
| Mody *et al.* 2019 | - | Yes | - | - | - | Quantitative |
| Moore *et al.* 2014 | - | Yes | - | Yes | Yes | Quantitative |
| Mospan *et al.* 2021 | Yes | Yes | - | - | - | Quantitative |
| Norman *et al*.2015 | Yes | Yes | Yes | Yes | - | Mixed Methods |
| O'Connell *et al*.2020 | - | - | Yes | - | - | Quantitative |
| Papineau *et al*. 2021 | Yes | - | - | - | - | Quantitative |
| Pelaccio *et al.* 2022 | Yes | - | Yes | - | - | Quantitative |
| Perraudin *et al.* 2011 | Yes | - | - | - | - | Quantitative |
| Rafie *et al.* 2019 | - | - | Yes | Yes | - | Qualitative |
| Rafie *et al*. 2022 (1) | - | Yes | Yes | - | - | Qualitative |
| Rafie *et al*. 2022 (2) | - | Yes | Yes | - | - | Quantitative |
| Raghunandan *et al*. 2021 | Yes | Yes | - | - | - | Quantitative |
| Rashid *et al.* 2022 | Yes | - | - | - | - | Quantitative |
| Reyes *et al.* 2020 | Yes | - | Yes | - | - | Quantitative |
| Rouleau *et al*. 2024 | Yes | Yes | - | Yes | - | Quantitative |
| Sadeq *et al*.2022 | Yes | - | - | - | - | Quantitative |
| Sadeq *et al.*2022 | Yes | - | - | - | - | Quantitative |
| Sandhu *et al.* 2018 | Yes | - | Yes | - | - | Quantitative |
| Seamon *et al*. 2020 | - | Yes | Yes | Yes | - | Quantitative |
| Spencer *et al*. 1992 | - | - | - | - | Yes | Quantitative |
| Stewart *et al.* 2009 | Yes | Yes | Yes | - | Yes | Quantitative |
| Stewart *et al*. 2018 | - | Yes | Yes | - | - | Quantitative |
| Stone *et al.* 2024 | Yes | Yes | - | Yes | - | Quantitative |
| Truter *et al.* 2018 | Yes | - | - | - | - | Quantitative |
| Ung *et al.* 2017 | Yes | Yes | - | - | - | Quantitative |
| Vracar *et al*. 2008 | - | - | - | - | Yes | Mixed Methods |
| Vu *et al*. 2019 | Yes | - | Yes | Yes | - | Quantitative |
| Waite *et al.* 2018 | Yes | - | - | - | - | Quantitative |
| Warner *et al*.2025 | Yes | Yes | Yes | Yes | Yes | Qualitative |
| Wirth *et al.* 2011 | Yes | Yes | Yes | Yes | - | Quantitative |
| Zhu *et al.* 2020 | Yes | - | - | - | Yes | Quantitative |

Legend: Study contributed to theme synthesis = Yes.

**Appendix 5: Quality Assessment with Mixed Methods Appraisal Tool (MMAT)**

| **Author, Year** | **S1.** | **S2** | **1.1** | **1.2** | **1.3** | **1.4** | **1.5** | **4.1** | **4.2** | **4.3** | **4.4** | | **4.5** | **5.1** | **5.2** | **5.3** | **5.4** | **5.5** | **MMAT flags** |
| --- | --- | --- | --- | --- | --- | --- | --- | --- | --- | --- | --- | --- | --- | --- | --- | --- | --- | --- | --- |
|  | * See description of MMAT methodological quality criteria below | | | | | | | | | | | | | | | | | |  |
| **Studies assessed using the MMAT mixed methods criteria** | | | | | | | | | | | | | | | | | | | |
| Amador-Fernández *et al.* 2024 | Yes | Yes | Yes | Yes | Yes | Yes | Yes | Yes | Can't tell | Yes | No | | Yes | Yes | Yes | Yes | Can't tell | Yes | 3 |
| Azad *et al.* 2024 | Yes | Yes | Yes | Yes | Yes | Yes | Yes | Yes | Yes | Yes | No | | Yes | Yes | Yes | Yes | Can't tell | Yes | 2 |
| Banawis *et al.* 2023 | Yes | Yes | Yes | Yes | Yes | Yes | Yes | Yes | Yes | Yes | Yes | |  | Yes | Yes | Yes | Can't tell | Yes | 1 |
| Booker *et al.* 2023 | Yes | Yes | Yes | Yes | Yes | Yes | Yes | Yes | No | Yes | No | | Yes | Yes | Yes | Yes | Yes | Yes | 2 |
| Grant *et al*. 2023 | Yes | Yes | Yes | Yes | Yes | Yes | Yes | Yes | No | Yes | No | | Yes | Yes | Yes | Yes | Yes | Yes | 2 |
| Grant *et al.* 2025 | Yes | Yes | Yes | Yes | Yes | Yes | Yes | Yes | Yes | Yes | Can't tell | | Yes | Yes | Yes | Yes | Yes | Yes | 1 |
| Irwin *et al.* 2019 | Yes | Yes | Yes | Yes | Yes | Yes | Yes | Yes | No | Yes | No | | Yes | Yes | Yes | Yes | Can't tell | Yes | 3 |
| Norman *et al*.2015 | Yes | Yes | Yes | Yes | Yes | Yes | Yes | Yes | Yes | Yes | No | | Yes | Yes | Yes | Yes | Yes | Yes | 1 |
| Vracar *et al*. 2008 | Yes | Yes | Yes | Yes | Yes | Yes | Yes | Yes | No | Yes | No | | Yes | Yes | Yes | Yes | Can't tell | Yes | 3 |
| **Studies assessed using the MMAT qualitative criteria** | | | | | | | | | | | | | | | | | | | |
| Amador-Fernández *et al.* 2023 | Yes | Yes | Yes | Yes | Yes | Yes | Yes | - | - | - | - | | - | - | - | - | - | - | 0 |
| Auta *et al.* 2016 | Yes | Yes | Yes | Yes | Yes | Yes | Yes | - | - | - | - | | - | - | - | - | - | - | 0 |
| BrilliantMoloto *et al*. 2024 | Yes | Yes | Yes | Yes | Yes | Yes | Yes | - | - | - | - | | - | - | - | - | - | - | 0 |
| Campi *et al.* 2024 | Yes | Yes | Yes | Yes | Yes | Yes | Yes | - | - | - | - | | - | - | - | - | - | - | 0 |
| Chuene *et al.* 2024 | Yes | Yes | Yes | Yes | Yes | Yes | Yes | - | - | - | - | | - | - | - | - | - | - | 0 |
| Dale *et al.* 2024 | Yes | Yes | Yes | Yes | Yes | Yes | Yes | - | - | - | - | | - | - | - | - | - | - | 0 |
| Famiyeh *et al*. 2019 | Yes | Yes | Yes | Yes | Yes | Yes | Yes | - | - | - | - | | - | - | - | - | - | - | 0 |
| Faruquee *et al.* 2019 | Yes | Yes | Yes | Yes | Yes | Yes | Yes | - | - | - | - | | - | - | - | - | - | - | 0 |
| Feehan *et al*. 2016 | Yes | Yes | Yes | Yes | Yes | Yes | Yes | - | - | - | - | | - | - | - | - | - | - | 0 |
| Gomez *et al.* 2020 | Yes | Yes | Yes | Yes | Yes | Yes | Yes | - | - | - | - | | - | - | - | - | - | - | 0 |
| Guirguis *et al.* 2014 | Yes | Yes | Yes | Yes | Yes | Yes | Yes | - | - | - | - | | - | - | - | - | - | - | 0 |
| Hatah *et al*. 2013 | Yes | Yes | Yes | Yes | Yes | Yes | Yes | - | - | - | - | | - | - | - | - | - | - | 0 |
| Herman et al. 2020 | Yes | Yes | Yes | Yes | Yes | Yes | Yes | - | - | - | - | | - | - | - | - | - | - | 0 |
| Hughes *et al*. 2003 | Yes | Yes | Yes | Yes | Yes | Yes | Yes | - | - | - | - | | - | - | - | - | - | - | 0 |
| Hughes *et al*. 2014 | Yes | Yes | Yes | Yes | Yes | Yes | Yes | - | - | - | - | | - | - | - | - | - | - | 0 |
| Koester *et al.* 2020 | Yes | Yes | Yes | Yes | Yes | Yes | Yes | - | - | - | - | | - | - | - | - | - | - | 0 |
| Lloyd *et al*. 2007 | Yes | Yes | Yes | Yes | Yes | Yes | Yes | - | - | - | - | | - | - | - | - | - | - | 0 |
| Makowsky *et al.* 2013 | Yes | Yes | Yes | Yes | Yes | Yes | Yes | - | - | - | - | | - | - | - | - | - | - | 0 |
| Mantzourani *et al.* 2023 | Yes | Yes | Yes | Yes | Yes | Yes | Yes | - | - | - | - | | - | - | - | - | - | - | 0 |
| Meredith *et al*. 2020 | Yes | Yes | Yes | Yes | Yes | Yes | Yes | - | - | - | - | | - | - | - | - | - | - | 0 |
| Rafie *et al.* 2019 | Yes | Yes | Yes | Yes | Yes | Yes | Yes | - | - | - | - | | - | - | - | - | - | - | 0 |
| Rafie *et al*. 2022 (1) | Yes | Yes | Yes | Yes | Yes | Yes | Yes | - | - | - | - | | - | - | - | - | - | - | 0 |
| Warner *et al*.2025 | Yes | Yes | Yes | Yes | Yes | Yes | Yes | - | - | - | - | | - | - | - | - | - | - | 0 |
| **Studies assessed using the MMAT quantitative descriptive criteria** | | | | | | | | | | | | | | | | | | | |
| AttardPizzuto *et al.* 2019 | Yes | Yes | - | - | - | - | - | Yes | No | Yes | No | | Yes | - | - | - | - | - | 2 |
| Auta *et al.* 2014 | Yes | Yes | - | - | - | - | - | No | No | Yes | Yes | | Yes | - | - | - | - | - | 2 |
| Baratta *et al.* 2025 | Yes | Yes | - | - | - | - | - | Yes | No | Yes | Yes | | Yes | - | - | - | - | - | 1 |
| Beahm *et al.* 2018 | Yes | Yes | - | - | - | - | - | Yes | Yes | Yes | Yes | | Yes | - | - | - | - | - | 0 |
| Cohen *et al.* 2024 | Yes | Yes | - | - | - | - | - | Yes | No | Yes | No | | Yes | - | - | - | - | - | 2 |
| Dahri *et al.* 2024 | Yes | Yes | - | - | - | - | - | Yes | No | Yes | No | | Yes | - | - | - | - | - | 2 |
| George *et al.* 2006 | Yes | Yes | - | - | - | - | - | Yes | No | Yes | No | | Yes | - | - | - | - | - | 2 |
| George *et al.* 2007 | Yes | Yes | - | - | - | - | - | Yes | Yes | Yes | Yes | | Yes | - | - | - | - | - | 0 |
| Gomez *et al*. 2022 | Yes | Yes | - | - | - | - | - | Yes | No | Yes | No | | Yes | - | - | - | - | - | 2 |
| Hoti *et al*. 2011 | Yes | Yes | - | - | - | - | - | Yes | No | No | No | | Yes | - | - | - | - | - | 3 |
| Hoti *et al*. 2013 | Yes | Yes | - | - | - | - | - | Yes | Can't tell | Yes | No | | Yes | - | - | - | - | - | 2 |
| Hunter *et al.* 2023 | Yes | Yes | - | - | - | - | - | Yes | No | Yes | No | | Yes | - | - | - | - | - | 2 |
| Karim *et al*. 2024 | Yes | Yes | - | - | - | - | - | Yes | Yes | Yes | No | | Yes | - | - | - | - | - | 1 |
| Lemay *et al.* 2025 | Yes | Yes | - | - | - | - | - | No | No | Yes | No | | Yes | - | - | - | - | - | 3 |
| Lewis *et al*. 2021 | Yes | Yes | - | - | - | - | - | Yes | No | Yes | No | | Yes | - | - | - | - | - | 2 |
| Lio *et al*. 2018 | Yes | Yes | - | - | - | - | - | No | No | Yes | No | | Yes | - | - | - | - | - | 3 |
| Lutz *et al.* 2021 | Yes | Yes | - | - | - | - | - | Yes | No | Yes | Yes | | Yes | - | - | - | - | - | 1 |
| Mansell *et al.*2015 | Yes | Yes | - | - | - | - | - | Yes | No | Yes | No | | Yes | - | - | - | - | - | 2 |
| Mhailan *et al.* 2022 | Yes | Yes | - | - | - | - | - | Yes | No | Yes | No | | Yes | - | - | - | - | - | 2 |
| Miszewska *et al.* 2022 | Yes | Yes | - | - | - | - | - | No | No | Yes | No | | Yes | - | - | - | - | - | 3 |
| Mody *et al.* 2019 | Yes | Yes | - | - | - | - | - | Yes | No | Can't tell | No | | Yes | - | - | - | - | - | 3 |
| Moore *et al.* 2014 | Yes | Yes | - | - | - | - | - | Yes | No | Yes | No | | Yes | - | - | - | - | - | 2 |
| Mospan *et al.* 2021 | Yes | Yes | - | - | - | - | - | Yes | No | Yes | Yes | | Yes | - | - | - | - | - | 1 |
| O'Connell *et al*.2020 | Yes | Yes | - | - | - | - | - | Yes | No | Yes | No | | Yes | - | - | - | - | - | 2 |
| Papineau *et al*. 2021 | Yes | Yes | - | - | - | - | - | Yes | No | Yes | No | | Yes | - | - | - | - | - | 2 |
| Pelaccio *et al.* 2022 | Yes | Yes | - | - | - | - | - | Yes | No | Yes | No | | Yes | - | - | - | - | - | 2 |
| Perraudin *et al.* 2011 | Yes | Yes | - | - | - | - | - | Yes | Yes | Yes | Yes | | Yes | - | - | - | - | - | 0 |
| Rafie *et al*. 2022 (2) | Yes | Yes | - | - | - | - | - | Yes | No | Yes | No | | Yes | - | - | - | - | - | 2 |
| Raghunandan *et al*. 2021 | Yes | Yes | - | - | - | - | - | Yes | Can't tell | Yes | No | | Yes | - | - | - | - | - | 2 |
| Rashid *et al.* 2022 | Yes | Yes | - | - | - | - | - | Yes | No | Yes | No | | Yes | - | - | - | - | - | 2 |
| Reyes *et al.* 2020 | Yes | Yes | - | - | - | - | - | Yes | No | Yes | No | | Yes | - | - | - | - | - | 2 |
| Rouleau *et al*. 2024 | Yes | Yes | - | - | - | - | - | Yes | No | Yes | No | | Yes | - | - | - | - | - | 2 |
| Sadeq *et al*.2022 | Yes | Yes | - | - | - | - | - | Yes | Can't tell | Yes | Yes | | Yes | - | - | - | - | - | 1 |
| Sandhu *et al.* 2018 | Yes | Yes | - | - | - | - | - | Yes | Can't tell | Yes | No | | Yes | - | - | - | - | - | 2 |
| Seamon *et al*. 2020 | Yes | Yes | - | - | - | - | - | Yes | No | Yes | No | | Yes | - | - | - | - | - | 2 |
| Sadeq *et al.*2022 | Yes | Yes | - | - | - | - | - | Yes | No | Yes | No | | Yes | - | - | - | - | - | 2 |
| Spencer *et al*. 1992 | Yes | Yes | - | - | - | - | - | Yes | Yes | Yes | Yes | | Yes | - | - | - | - | - | 0 |
| Stewart *et al.* 2009 | Yes | Yes | - | - | - | - | - | Yes | No | Yes | No | | Yes | - | - | - | - | - | 2 |
| Stewart *et al*. 2018 | Yes | Yes | - | - | - | - | - | Yes | No | Yes | No | | Yes | - | - | - | - | - | 2 |
| Stone *et al.* 2024 | Yes | Yes | - | - | - | - | - | Yes | No | Yes | No | | Yes | - | - | - | - | - | 2 |
| Truter *et al.* 2018 | Yes | Yes | - | - | - | - | - | Yes | No | Yes | No | | Yes | - | - | - | - | - | 2 |
| Ung *et al.* 2017 | Yes | Yes | - | - | - | - | - | Yes | No | Yes | No | | Yes | - | - | - | - | - | 2 |
| Vu *et al*. 2019 | Yes | Yes | - | - | - | - | - | Yes | No | Yes | No | | Yes | - | - | - | - | - | 2 |
| Waite *et al.* 2018 | Yes | Yes | - | - | - | - | - | Yes | No | Yes | No | | Yes | - | - | - | - | - | 2 |
| Wirth *et al.* 2011 | Yes | Yes | - | - | - | - | - | Yes | Yes | Yes | Yes | | Yes | - | - | - | - | - | 0 |
| Zhu *et al.* 2020 | Yes | Yes | - | - | - | - | - | Yes | No | Yes | No | | Yes | - | - | - | - | - | 2 |
| *** Legend: MMAT methodological quality criteria**  S1. Are there clear research questions? | | | | | | | | | | | |  |  |  |  |  |  |  |  |
| S2. Do the collected data allow to address the research questions? | | | | | | | | | | | |  |  |  |  |  |  |  |  |
| 1.1. Is the qualitative approach appropriate to answer the research question? | | | | | | | | | | | |  |  |  |  |  |  |  |  |
| 1.2. Are the qualitative data collection methods adequate to address the research question? | | | | | | | | | | | |  |  |  |  |  |  |  |  |
| 1.3. Are the findings adequately derived from the data? | | | | | | | | | | | |  |  |  |  |  |  |  |  |
| 1.4. Is the interpretation of results sufficiently substantiated by data? | | | | | | | | | | | |  |  |  |  |  |  |  |  |
| 1.5. Is there coherence between qualitative data sources, collection, analysis and interpretation? | | | | | | | | | | | |  |  |  |  |  |  |  |  |
| 4.1. Is the sampling strategy relevant to address the research question? | | | | | | | | | | | |  |  |  |  |  |  |  |  |
| 4.2. Is the sample representative of the target population? | | | | | | | | | | | |  |  |  |  |  |  |  |  |
| 4.3. Are the measurements appropriate? | | | | | | | | | | | |  |  |  |  |  |  |  |  |
| 4.4. Is the risk of nonresponse bias low? | | | | | | | | | | | |  |  |  |  |  |  |  |  |
| 4.5. Is the statistical analysis appropriate to answer the research question? | | | | | | | | | | | |  |  |  |  |  |  |  |  |
| 5.1. Is there an adequate rationale for using a mixed methods design to address the research question? | | | | | | | | | | | |  |  |  |  |  |  |  |  |
| 5.2. Are the different components of the study effectively integrated to answer the research question? | | | | | | | | | | | |  |  |  |  |  |  |  |  |
| 5.3. Are the outputs of the integration of qualitative and quantitative components adequately interpreted? | | | | | | | | | | | |  |  |  |  |  |  |  |  |
| 5.4. Are divergences and inconsistencies between quantitative and qualitative results adequately addressed? | | | | | | | | | | | |  |  |  |  |  |  |  |  |
| 5.5. Do the different components of the study adhere to the quality criteria of each tradition of the methods involved? | | | | | | | | | | | |  |  |  |  |  |  |  |  |

**Appendix 6: Distribution of analytical theme codes across implementation stages**

| **Analytical Theme** | **Pre** | **Peri** | **Post** | **Total** |
| --- | --- | --- | --- | --- |
| 1. Support, motivation and clinical readiness | 281 | 35 | 250 | **566** |
| 2. Structural, policy and organisational conditions shaping feasibility | 90 | 27 | 169 | **286** |
| 3. Patient-centred access, continuity, and therapeutic relationship | 118 | 15 | 117 | **250** |
| 4. Moral responsibility, risk, and safe practice boundaries | 72 | 16 | 79 | **166** |
| 5. Professional identity, autonomy, and legitimacy | 77 | 6 | 58 | **141** |
| **Total** | **638** | **98** | **673** | **1409** |
